# Heritable modifiers of the tumor microenvironment influence nanoparticle uptake, distribution and response to photothermal therapy

**DOI:** 10.7150/thno.41171

**Published:** 2020-04-06

**Authors:** Gayatri Sharma, Jaidip M. Jagtap, Abdul K. Parchur, Venkateswara R. Gogineni, Sophia Ran, Carmen Bergom, Sarah B. White, Michael J. Flister, Amit Joshi

**Affiliations:** 1 Department of Biomedical Engineering, Medical College of Wisconsin, Milwaukee, WI, USA; 2 Department of Radiology, Medical College of Wisconsin, Milwaukee, WI, USA; 3 Simmons Cancer Institute, Southern Illinois University School of Medicine, Springfield, IL, USA; 4 Department of Medical Microbiology, Immunology, and Cell Biology, Southern Illinois University School of Medicine, Springfield, IL, USA; 5 Department of Radiation Oncology, Medical College of Wisconsin, Milwaukee, WI, USA; 6 Department of Physiology, Medical College of Wisconsin, Milwaukee, WI, USA; 7 Genomic Sciences and Precision Medicine Center, Medical College of Wisconsin, Milwaukee, WI 53226, USA

**Keywords:** Nanoparticles, Photothermal, Tumor Microenvironment, Breast Cancer, Tumor Vasculature

## Abstract

We report the impact of notch-DLL4-based hereditary vascular heterogeneities on the enhanced permeation and retention (EPR) effect and plasmonic photothermal therapy response in tumors.

**Methods**: We generated two consomic rat strains with differing DLL4 expression on 3^rd^ chromosome. These strains were based on immunocompromised Salt-sensitive or SS^IL2Rγ-^ (DLL4-high) and SS.BN3^IL2Rγ-^ (DLL4-low) rats with 3rd chromosome substituted from Brown Norway rat. We further constructed three novel SS.BN3^IL2Rγ-^ congenic strains by introgressing varying segments of BN chromosome 3 into the parental SS^IL2Rγ-^ strain to localize the role of SS^IL2Rγ-^ DLL4 on tumor EPR effect with precision. We synthesized multimodal theranostic nanoparticles (TNPs) based on Au-nanorods which provide magnetic resonance imaging (MRI), X-ray, and optical contrasts to assess image guided PTT response and quantify host specific therapy response differences in tumors orthotopically xenografted in DLL4-high and -low strains. We tested recovery of therapy sensitivity of PTT resistant strains by employing anti-DLL4 conjugated TNPs in two triple negative breast cancer tumor xenografts.

**Results**: Host strains with high DLL4 allele demonstrated slightly increased tumor nanoparticle uptake but consistently developed photothermal therapy resistance compared to tumors in host strains with low DLL4 allele. Tumor micro-environment with low DLL4 expression altered the geographic distribution of nanoparticles towards closer proximity with vasculature which improved efficacy of PTT in spite of lower overall TNP uptake. Targeting TNPs to tumor endothelium via anti-DLL4 antibody conjugation improved therapy sensitivity in high DLL4 allele hosts for two triple negative human breast cancer xenografts.

**Conclusions**: Inherited DLL4 expression modulates EPR effects in tumors, and molecular targeting of endothelial DLL4 via nanoparticles is an effective personalized nanomedicine strategy.

## Introduction

Nanomedicine leverages the tunable pharmacokinetics and bio-distribution of ~100 nm size therapeutic nanoparticles (NPs) to solid tumors, promising dramatically higher tumor doses with reduced off-target uptake 1, 2. However, only a limited number of nanomedicine agents such as Abraxane^TM^ (Albumin NP bound Paclitaxel) and Doxil^TM^ (liposomal NP doxorubicin) have gained widespread use in clinic for advanced solid tumors, with a moderate improvement in progression-free survival for advanced breast cancer patients 3, 4. Further nanomedicine benefits are less evident in expanded clinical trials 2, 5, 6. Even molecularly targeted nanomedicine, such as PEGylated polymeric nanoparticle Docetaxel 1 or HER2-targeted liposomal Doxorubicin, have failed initial or phase II clinical trials 7, possibly due to patient and tumor heterogeneity in nanomedicine uptake, distribution, therapeutic responses and clearance due to mononuclear phagocyte system 8.

Alternate near-infrared (NIR) light-triggered gold nanoparticle (AuNP)-mediated photo-thermal therapies (PTT), rely on spatiotemporally controlled thermal ablation of tumors with low power NIR light illumination. These therapies are immune to known drug resistance mechanisms and have demonstrated exceptional efficacy in mouse models of aggressive breast cancer with negligible off-target effects 9. AuNP-mediated PTT is under clinical investigation for multiple solid tumors 10-14, but the widespread clinical translation might be hampered by the same factors that are applicable to other targeted medicine approaches - i.e., the inability to accommodate for tumor heterogeneity and non-uniform therapy response 15, 16. Furthermore, despite the evolution of personalized precision medicine 17, nanomedicine strategies have yet to be tailored to individual patient features, such as heterogeneity in enhanced permeability and retention (EPR) 18-22, to achieve the long-predicted enhancements in efficacy.

There is increasing evidence about the role of inherited or germline genetic modifiers in TME heterogeneity and EPR effects, yet the underlying drivers have remained largely unknown because a systematic approach to study them did not exist 23. To overcome this issue, we recently developed the Consomic Xenograft Model (CXM) as the first strategy for mapping heritable modifiers of TME heterogeneity 24. In CXM, human breast cancer cells are orthotopically implanted into genetically-engineered consomic xenograft host strains, which are derived from two parental strains with different susceptibilities to breast cancer. Because the host strain backgrounds are different, whereas the inoculated tumor cells are the same, any phenotypic variation is due to TME modifier(s) on the substituted chromosome and can be further localized by congenic mapping. Using the CXM strategy, we recently identified a vascular-specific delta-like 4 (*DLL4*) modifier allele on rat chromosome 3 (RNO3) that functioned as a heritable host TME modifier of EPR 23-26. Notably, DLL4 is a master regulator of angiogenic vascular patterning 27-35 and inhibition of DLL4 attenuates tumor growth and progression by eliciting nonproductive angiogenesis 28, 31-35, yet the explicit role of DLL4 in EPR and its influence on NP delivery and efficacy remains untested. Even less understood is the potential impact that inheritance of functionally distinct *DLL4* alleles might have on the patient-to-patient variability in response to NP therapy, which ultimately could lead to the failure of NPs in clinical trials.

Here, we used two CXM strains, SS^IL2Rγ-^ (DLL4-high) and SS.BN3^IL2Rγ-^ (DLL4-low) 24, as well as three congenic xenograft strains, JQ^IL2Rγ-^, ND^IL2Rγ-^, and MX^IL2Rγ-^ to assess the impact of germline TME vascular heterogeneity on NP delivery and PTT efficacy. We used novel multimodal theranostic nanoparticles (TNPs) composed of Au nanorods (AuNRs) coated with stable Gd(III)-oxide epilayers, which provide magnetic resonance imaging (MRI), X-ray, and photothermal contrast in a sub-100nm geometry 36. MRI and PTT with these TNPs revealed that although cross-sectional contrasts can reveal optimal NP uptake in tumors, it is the inherited microvascular distribution patterns, and not the overall NP uptake, which govern the efficacy of NP-mediated PTT. Molecularly targeting DLL4 expression on tumor vasculature with antibody conjugated TNPs recovered PTT sensitivity in otherwise therapy resistant SS^IL2Rγ-^ (DLL4-high) strain. Collectively, these data are the first to demonstrate that inherited TME heterogeneity dramatically impacts the efficacy of PTT, which should be accounted for and targeted in the development of future personalized nanomedicine strategies.

## Results

**Figure 1:**
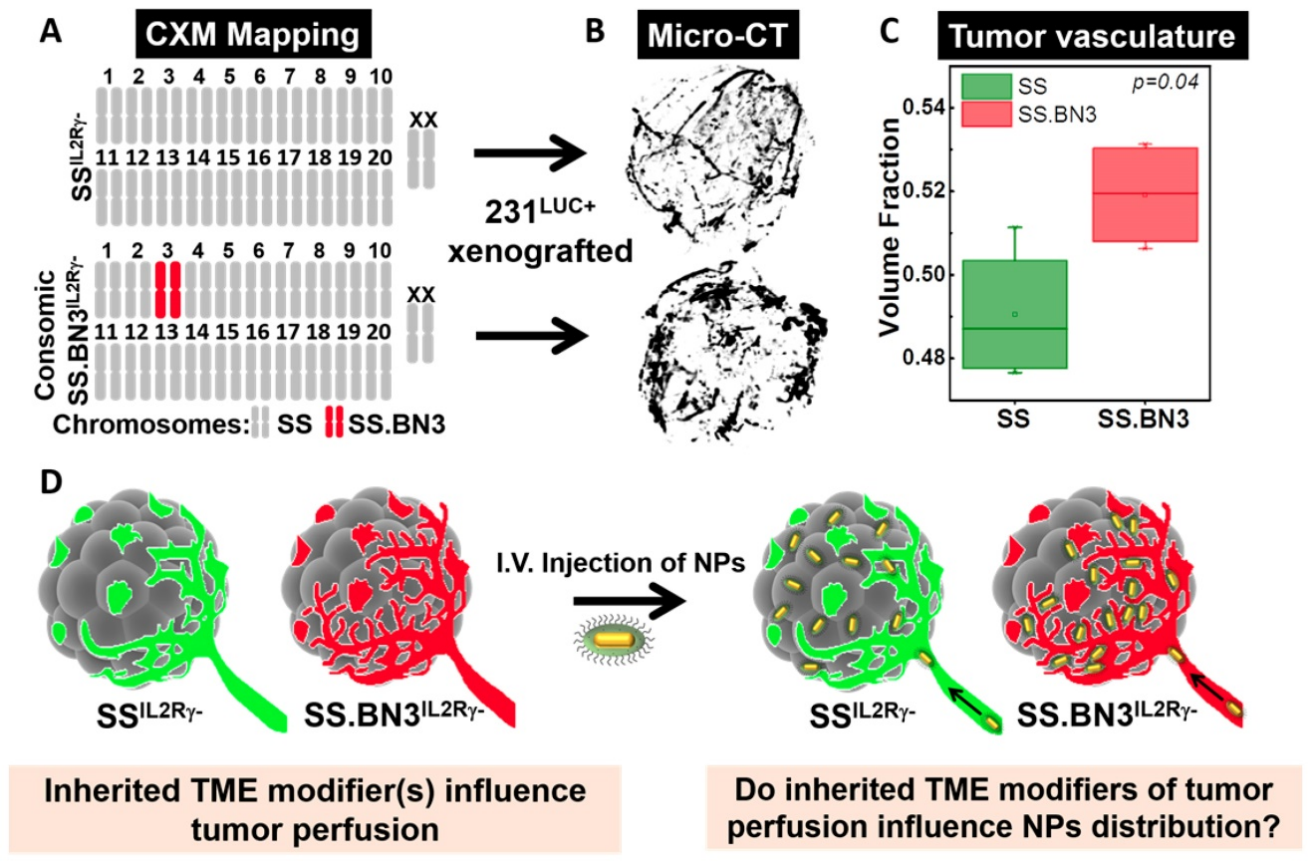
Schematic representation of the CXM and experimental details. (A) Schematic representation of the SS and SS.BN3 genomes modified by TALEN-mediated editing of the IL2Rγ gene. It represents chromosome that is derived from SS (grey) or BN3 (red). The genetic differences between SS^IL2Rγ-^ and SS.BN3^IL2Rγ-^ are due to inheritance of chromosome 3 from the SS or BN rats. (B) The luciferase-expressing MDA-MB-231 breast cancer cells (231^LUC+^) were orthotopically implanted in the mammary fat pad of SS^IL2Rγ-^ and SS.BN3^IL2Rγ-^ rats. MicroCT 3D volume rendering of vessel network in tumors generated using X-ray MicroCT data of 231^LUC+^ breast tumors implanted in SS^IL2Rγ-^ (n = 4) and SS.BN3^IL2Rγ-^ (n = 4) rats. (C) Vascular volume fraction in SS^IL2Rγ-^ and SS.BN3^IL2Rγ-^ rat tumors (*P* = 0.04, t-test). (D) Ten days after tumor development AuNRs/TNPs were injected intravenously. Since same 231^LUC+^ cells (gray) were implanted in both the strains, different distribution of AuNRs/TNPs can be attributed to differences in the SS^IL2Rγ-^ (green) and SS.BN3^IL2Rγ-^ (red) microenvironments (IV: Intravenous injection; TME: Tumor Microenvironment, NPs: Nanoparticles).

**CXM models generate distinct vascular patterning in identical triple negative breast cancer tumors**. The previously characterized CXM models of TME vascular heterogeneity, SS^IL2Rγ-^ (DLL4-high) and SS.BN3^IL2Rγ-^ (DLL4-low) 24, 25, were used to determine the impact of tumor vascular organization on TNP uptake, distribution, and PTT response (**Figure 1A**). We verified the vascular morphology differences in these strains first *via ex vivo* micro-CT imaging in intact tumors, and then *via* immunofluorescence microscopy in tumor sections. The micro-CT imaging using microfil contrast agent enabled the quantification of the volume fraction which is defined as the ratio of the vessel volume to the tumor volume of triple negative breast cancer tumors implanted in SS^IL2Rγ-^ and SS.BN3^IL2Rγ-^ hosts. Representative 3D volume reconstruction of vessel network in tumors from SS^IL2Rγ-^ and SS.BN3^IL2Rγ-^ hosts indicated increased irregular sprouting of vessels with a highly tortuous pattern of branching in SS.BN3^IL2Rγ-^ tumors is depicted in **Figure 1B**. The average volume fraction was higher in SS.BN3^IL2Rγ-^ hosted tumors as compared with SS^IL2Rγ-^ hosted tumors (**Figure 1C**, *P*<0.04). These results correlated well with histological findings (which will be discussed later) and supported the use of our SS^IL2Rγ-^ and SS.BN3^IL2Rγ-^ CXM model in subsequent investigations to determine the role of vascular TME heterogeneity on TNP uptake and PTT response after systemic delivery (**Figure 1D**).

**Figure 2:**
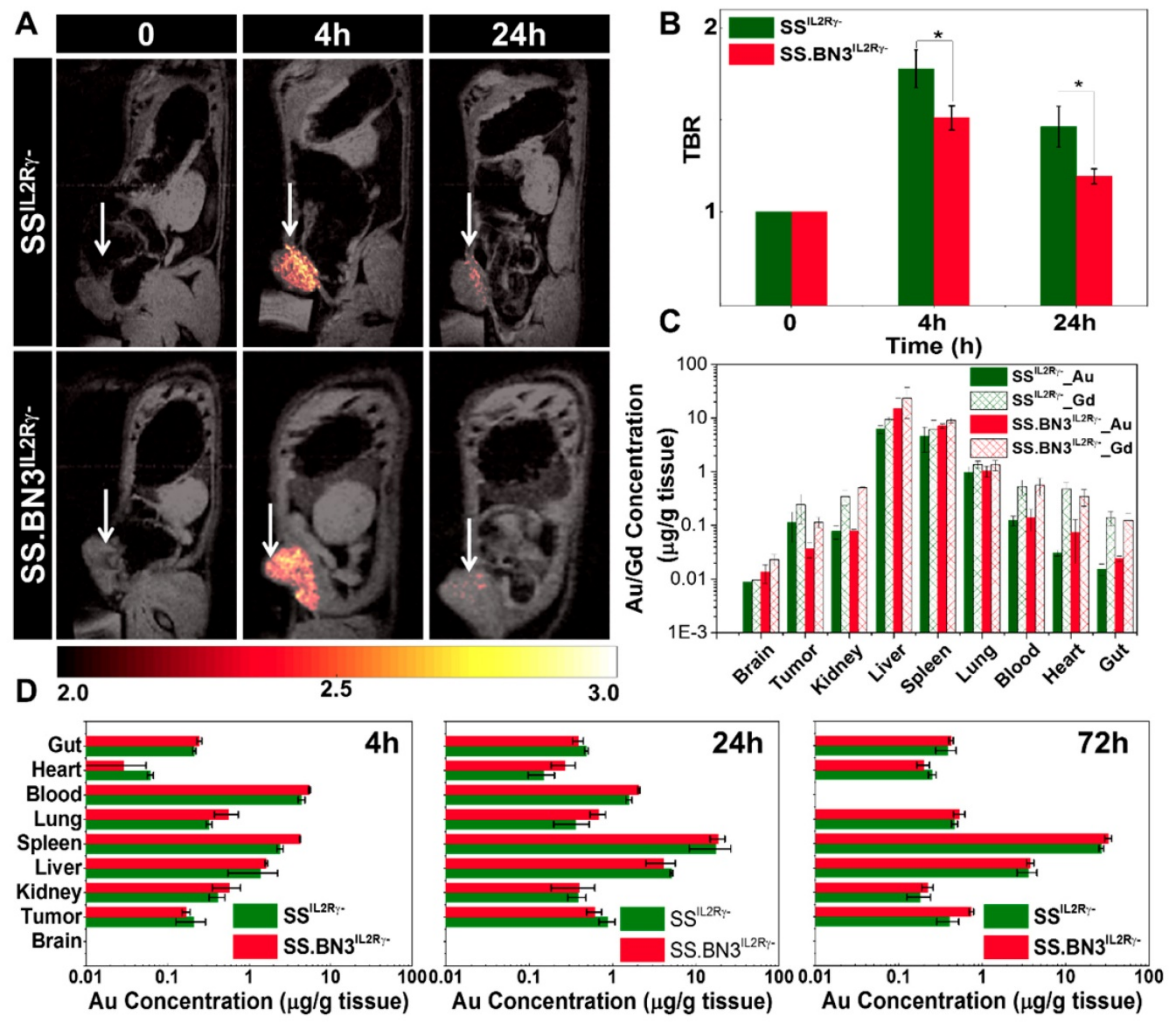
T_1_ contrast MR imaging and bio distribution by ICP-MS after systemic delivery of TNPs in 231^LUC+^ tumors implanted in SS^IL2Rγ-^ and SS.BN3^IL2Rγ-^ rats. (A) Monitoring *in vivo* T_1_-weighted MR images of SS^IL2Rγ-^ and SS.BN3^IL2Rγ-^ rats with 231^LUC+^ tumors for pre- (0 h), post-4 h, and post-24 h systemic injection. The tumor is marked with white arrow. (B) TBR enhancement comparison of 231^LUC+^ tumors between SS^ IL2Rγ-^ and SS.BN3^ IL2Rγ-^ rats (n = 3). (C) Bio distributions of TNPs and AuNRs in 231^LUC+^ implanted SS^ IL2Rγ-^ and SS.BN3^ IL2Rγ-^ rats were analyzed by ICP-MS. Mean and standard deviation of Au and Gd content in different organs including tumor was determined after post- 24 h of systemic injection. Mean and standard deviation of Au content in the organs determined after (D) 4 h, (E) 24 h and (F) 72 h of systemic injection of AuNRs. The Au and Gd content in tumors in μg of metal/g of wet tissue are plotted as a log10 scale for visual clarity of values in organs with low nanoparticle content. All the data are shown as the mean±s.e.

**Assessment of gadolinium-gold TNP biodistribution in SS.BN3^IL2Rγ-^ (DLL4-low) and SS^IL2Rγ-^ (DLL4-high) rats.** We recently developed a novel gadolinium-gold TNP (size 75 nm and charge +7.6 mV) that enables PTT and analysis of NP bio-distribution by MR imaging and inductively coupled plasma mass spectrometry (ICP-MS) using a single TNP nanoconstruct. Prior to testing the TNP efficacy in tumor-bearing SS.BN3^IL2Rγ-^ and SS^IL2Rγ-^ rats, a MRI study was performed (n = 3 rats per strain) to identify the optimal time point for PTT after systemic injection of TNPs and to detect if there were differences in overall TNP uptake in the tumors on the DLL4-high and DLL4-low host strains. T1-weighted MR imaging confirms the optimum tumor-to-background ratio (TBR) enhancement at post-4 h was observed compared to pre- and post- 24 h after systemic injection of TNPs as depicted in **Figure 2A-B**, a slightly higher T1- contrast in SS^IL2Rγ-^ rats as compared to SS.BN3^IL2Rγ-^ rats was observed. Immediately post- 24 h MR imaging animals were euthanized and ICP-MS was performed to validate the presence Au and Gd in per gram of different tissues (brain, tumor, kidney, liver, spleen, lung, blood, heart, and gut) as depicted in **Figure 2C**, confirming slightly higher amount of Gd (II) in SS^IL2Rγ-^ rats' tumor compared to SS.BN3^IL2Rγ-^ rats', which supports T1-weighted MR imaging results. Further, to understand the distribution of NP of different size and charge at different time point ICP-MS analysis was performed at post- 4 h, 24 h, and 72 h after systemic injection of AuNRs (10 nm by 40 nm and charge -9 mV) for different tissues (brain, tumor, kidney, liver, spleen, lung, blood, heart, and gut) depicted **Figure 2D.** The Au content in the tumor after post-24 h of systemic injection of AuNRs is higher than TNPs which could be due to impact of size variation. As we have previously, reported that size variation strongly impacts uptake of Au nanoparticle in MDA-MB-231 tumors 37. In all other organs, a similar NPs distribution was observed like TNP's ICP-MS study (**Figure 2C**) up to 24 h post injection, whereas there is a slight decrease in Au/g of tissue with slightly higher error bar in SS^IL2Rγ-^ rats as compared with SS.BN3^IL2Rγ-^ rats at 72 h post injection. These results suggest that SS^IL2Rγ-^ tumors retain higher overall levels of NP at the optimal 4 h time point for effective PTT.

**Figure 3:**
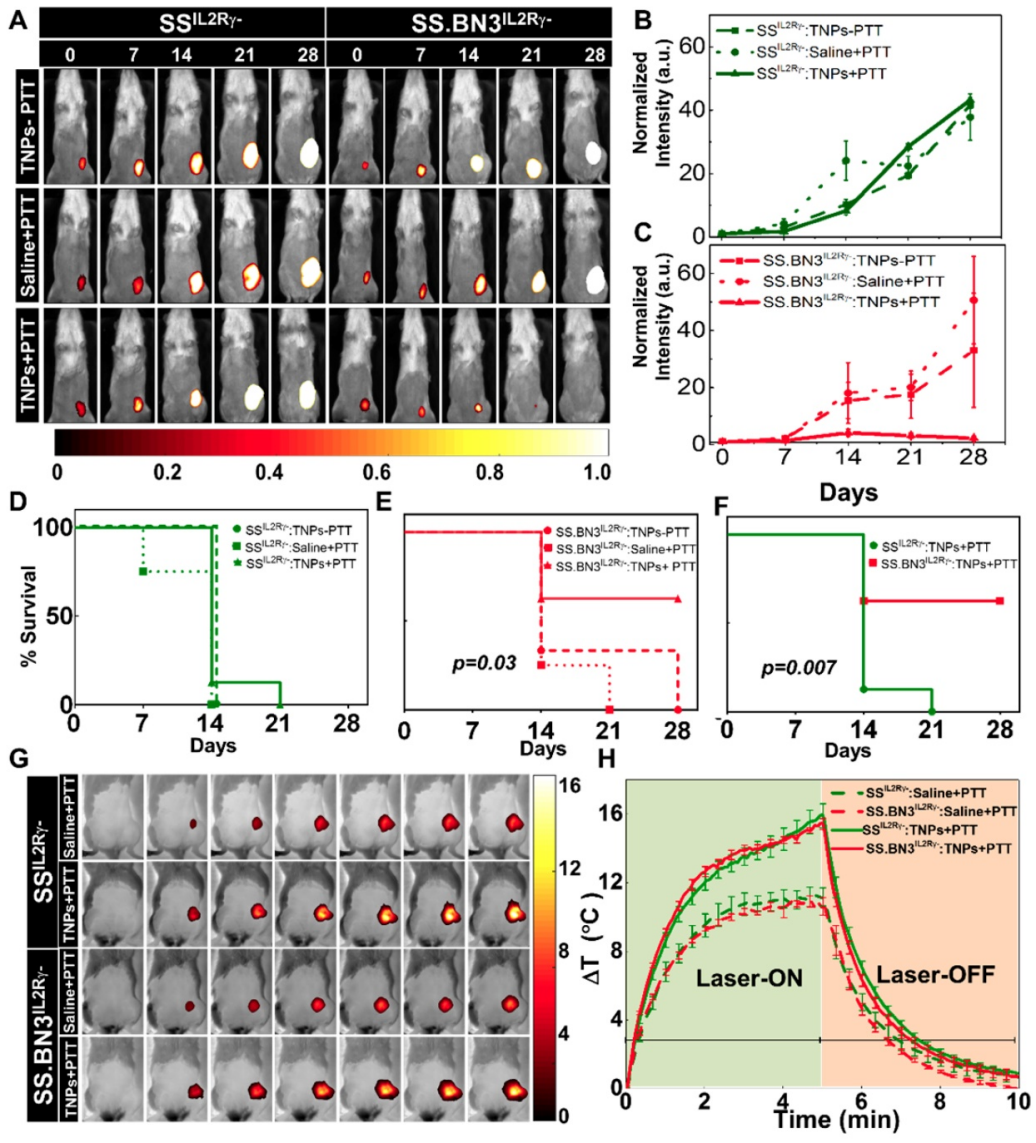
Evaluation of tumor response to photothermal therapy by bioluminescence imaging and Temperature kinetics during photothermal therapy (A) Representative images of saline and TNPs treated SS^IL2Rγ-^ and SS.BN3^IL2Rγ-^ rats. Saline and laser treated SS^IL2Rγ-^ (n=4) and SS.BN3^IL2Rγ-^(n=4) rats experienced a continuous increase. TNPs injected SS^IL2Rγ-^(n=4) and SS.BN3^IL2Rγ-^ (n=4) rats without laser treatment experienced a continuous increase of bioluminescence. SS^IL2Rγ-^ (n=8) rats experienced an increase in bioluminescence while SS.BN3^IL2Rγ-^ (n=8) rats experienced complete loss of tumor when treated with laser. Rats were followed for 4 weeks after treatment. The luciferase signal in all groups of (B) SS^IL2Rγ-^ and (C) SS.BN3^IL2Rγ-^rats was normalized to the signal before treatment. Survival curves of tumor-bearing (D) SS^IL2Rγ-^ and (E) SS.BN3^IL2Rγ-^ treated with TNPs only and saline solution or TNPs followed by 808-nm NIR laser irradiation for 5 min with 1.65 W/cm^2^ laser power covering ~2 cm^2^ areas. SS.BN3^IL2Rγ-^ rats treated with TNPs and laser responded better, and trend difference was statistically significant (*P* = 0.03, Wilcoxon Test). (F) Survival curves of tumor-bearing SS^IL2Rγ-^ and SS.BN3^IL2Rγ-^ treated with TNPs and laser vary significantly (*P* = 0.007, Wilcoxon Test). (G) FLIR thermal images of 231^LUC+^ implanted SS^IL2Rγ-^ and SS.BN3^IL2Rγ-^ rats acquired after 24 h of saline and TNPs systemic injection, irradiated by an 808-nm NIR laser for 5 min with ~1.65 W/cm^2^ laser power covering ~2 cm^2^ areas and its temperature kinetics is depicted in (H) followed by cooling for 5 min. Temperature change (ΔT) is calculated by subtracting the surface temperature at the starting time point (37°C). All the data are shown as the mean±s.e.

**PTT inhibits tumor growth in SS.BN3^IL2Rγ-^ (DLL4-low) but not SS^IL2Rγ-^ (DLL4-high).** To assess the impact of the TME vascular heterogeneity on PTT, SS.BN3^IL2Rγ-^ and SS^IL2Rγ-^ rats aged 4-6 weeks were orthotopically implanted with 6 x 10^6^ of luciferase expressing MDA-MB-231(231^LUC+^) triple negative breast cancer cells in the inguinal mammary fat pad. Rats were randomized into three groups per strain: group 1 (saline+laser; n = 4), group 2 (TNPs without laser; n = 4), and group 3 (TNPs+laser; n = 8). Analysis of tumor growth inhibition (TGI) by bioluminescent imaging revealed strong TGI in the triple negative breast cancer cells implanted in SS.BN3^IL2Rγ-^ rats treated by PTT with TNPs (**Figure 3A-C**), whereas no TGI was observed after identical treatment of triple negative breast cancer cells implanted in SS^IL2Rγ-^ rats (**Figure 3B**). The impact of TME vascular heterogeneity on TGI by PTT with TNPs was further reflected by a significant difference in survival of SS.BN3^IL2Rγ-^ rats (63%) compared with the SS^IL2Rγ-^ (0%; *P* = 0.007) at 21 days' post-implantation (**Figure 3D-F**). As depicted in **Figure 3G-H**, nearly identical ablative temperatures were achieved in tumors grown in the SS.BN3^IL2Rγ-^ (Δ15.4°C) and SS^IL2Rγ-^ (Δ16°C), indicating that differences in ablative temperature did not impact the efficacy of PTT with TNPs between the two strains. Importantly, the efficacy of PTT with the novel gadolinium-gold TNPs was not an artifact of TNPs characteristics (~70 nm size, +7.2 mV zeta potential; **Figure S1**), as identical results were obtained in an identical experiment using conventional AuNRs (10 nm x 40 nm size, -9 mV zeta potential, Nanopartz, Inc.) (**Figure S2-S4**). Thus, these data collectively demonstrate that the inherited TME vascular heterogeneity between the SS^IL2Rγ-^ (DLL4-high) and SS.BN3^IL2Rγ-^ (DLL4-low) rats 24-26 strongly modifies the therapeutic efficacy of PTT with TNPs, despite the implanted triple negative breast cancer cells being identical.

**Figure 4:**
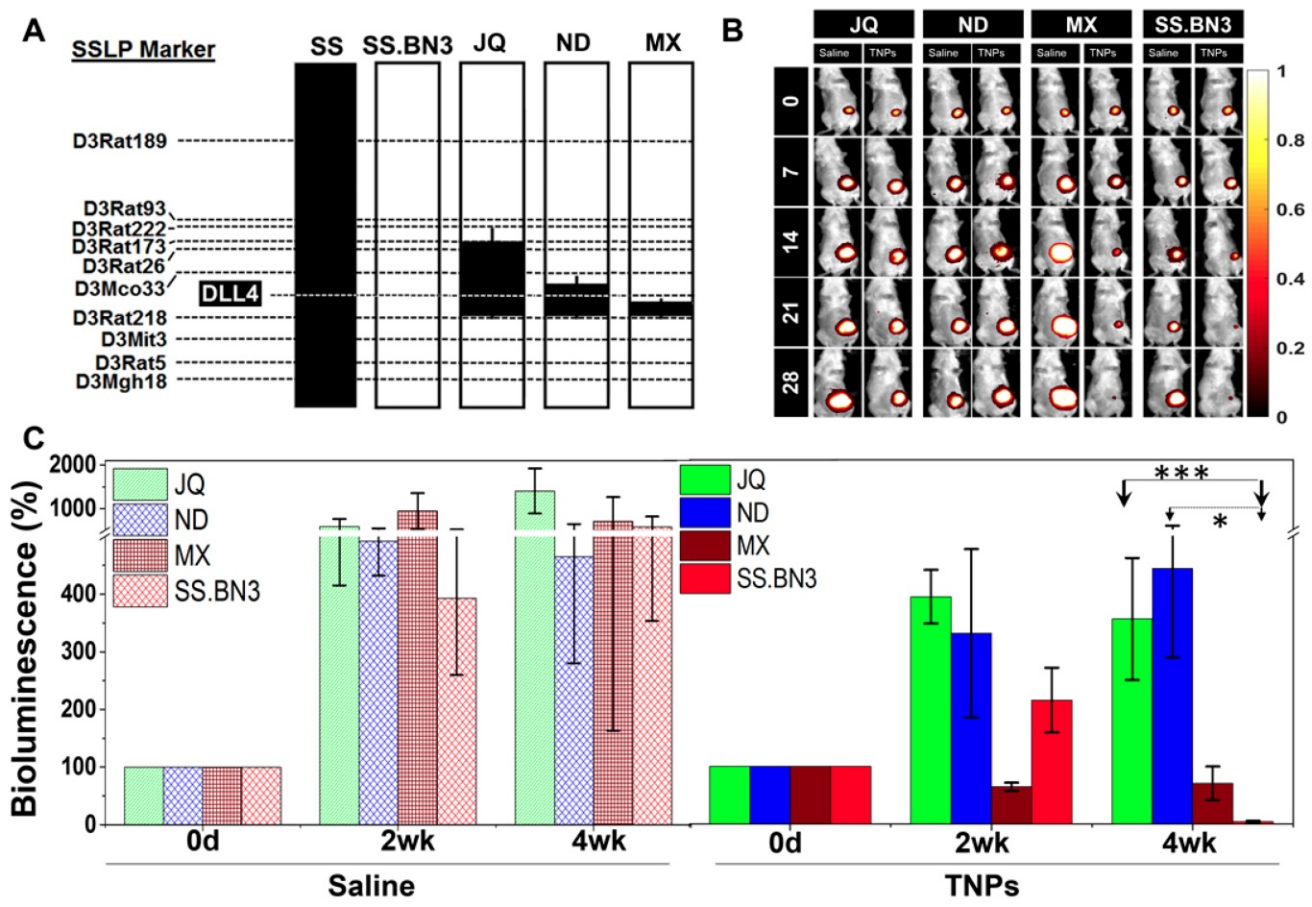
Evaluation of SS.BN3^IL2Rγ-^ congenic strains response to photothermal therapy. (A) Schematic representation of the SS.BN3^IL2Rγ-^ congenic strains that were generated by introgressing segments of BN chromosome 3 (black) into the genetic background of the parental SS^IL2Rγ-^ strain (white) by marker-assisted breeding. Thin black bars represent confidence intervals, which are chromosomal regions that could be BN or SS. (B) Representative images of saline and TNPs treated JQ^IL2Rγ-^, ND^IL2Rγ-^, MX^IL2Rγ-^ and SS.BN3^IL2Rγ-^ rats. (C) Bioluminescence (%) with standard deviation. TNPs and laser treated injected JQ^IL2Rγ-^ (n=6) and ND^IL2Rγ-^ (n=6) experienced increase in bioluminescence while MX^IL2Rγ-^ (n=6) and SS.BN3^IL2Rγ-^ (n=4) rats experienced complete loss of tumor. Saline and laser treated JQ^IL2Rγ-^ (n=3) and ND^IL2Rγ-^ (n=3) MX^IL2Rγ-^ (n=4) and SS.BN3^IL2Rγ-^ (n=3) experienced increase in bioluminescence. Rats were followed for 4 weeks after treatment. * Significant difference in bioluminescence of TNPs treated JQ^IL2Rγ-^ (*P*<0.001, t-test) and ND^ IL2Rγ-^ (*P*<0.05, t-test) with respect to SS.BN3^ IL2Rγ-.^ All the data are shown as the mean±s.e.

**Congenic mapping of the *DLL4* modifier locus of TME vascular heterogeneity and PTT efficacy.** Previously, we localized inherited modifier(s) of TME vascular heterogeneity to RNO3 by CXM mapping 24, which were then narrowed by congenic mapping to a 36Mb locus containing *DLL4* alleles with distinct vascular expression patterns in the SS.BN3^IL2Rγ-^ consomic (DLL4-low) and SS^IL2Rγ-^ (DLL4-high) rat strains 25. Although the SS.BN3^IL2Rγ-^ consomic data suggest that the DLL4 modifier allele impacts the efficacy of PTT with TNPs (**Figure 3 and Figure S2-4**), many other candidate alleles exist on chromosome 3 and therefore could account for the differences observed in PTT efficacy between SS.BN3^IL2Rγ-^ and SS^IL2Rγ-^. To address this issue, we constructed three novel SS.BN3^IL2Rγ-^ congenic xenograft host strains (JQ^IL2Rγ-^, ND^IL2Rγ-^, and MX^IL2Rγ-^) by introgressing segments of BN chromosome 3 (black) into the genetic background of the parental SS^IL2Rγ-^ strain (**Figure 4A**). The exclusion congenic mapping localized a 7.9 Mb (chr3:108, 855, 637 - 116, 715, 770) candidate region that was associated with inherited tumor vascular heterogeneity and contained the *DLL4* locus (**Figure 4A**). After orthotopic implantation of 231^LUC+^ triple negative breast cancer cells (6 x 10^6^) into the congenic strains (JQ^IL2Rγ-^, ND^IL2Rγ-^, and MX^IL2Rγ-^) and the parental SS.BN3^IL2Rγ-^ consomic strain, animals were injected with TNPs and PTT was performed as described previously. SS.BN3^IL2Rγ^ and MX^IL2Rγ-^ strains revealed significantly greater TGI as compared with the JQ^IL2Rγ-^ and ND^IL2Rγ-^ strains (**Figure 4B-C**). A multivariable linear regression model was used to analyze the relationship between the TGI observations with covariates time-post-therapy and strain. Coefficients for strains JQ^IL2Rγ-^ (*P*<0.001) and ND^IL2Rγ-^ strains (*P*<0.05) were statistically significant, indicating that these strains inheriting the *DLL4* locus from SS^IL2Rγ-^ (DLL4-high) were different from the baseline SS.BN3^IL2Rγ^ (DLL4-low) strain after adjusting for other covariates. Collectively, this data demonstrates that the congenic strains with the inherited *DLL4*-high allele and TME vascular heterogeneity are far more resistant to PTT with TNPs compared with those that inherited the *DLL4-*low allele.

**Figure 5:**
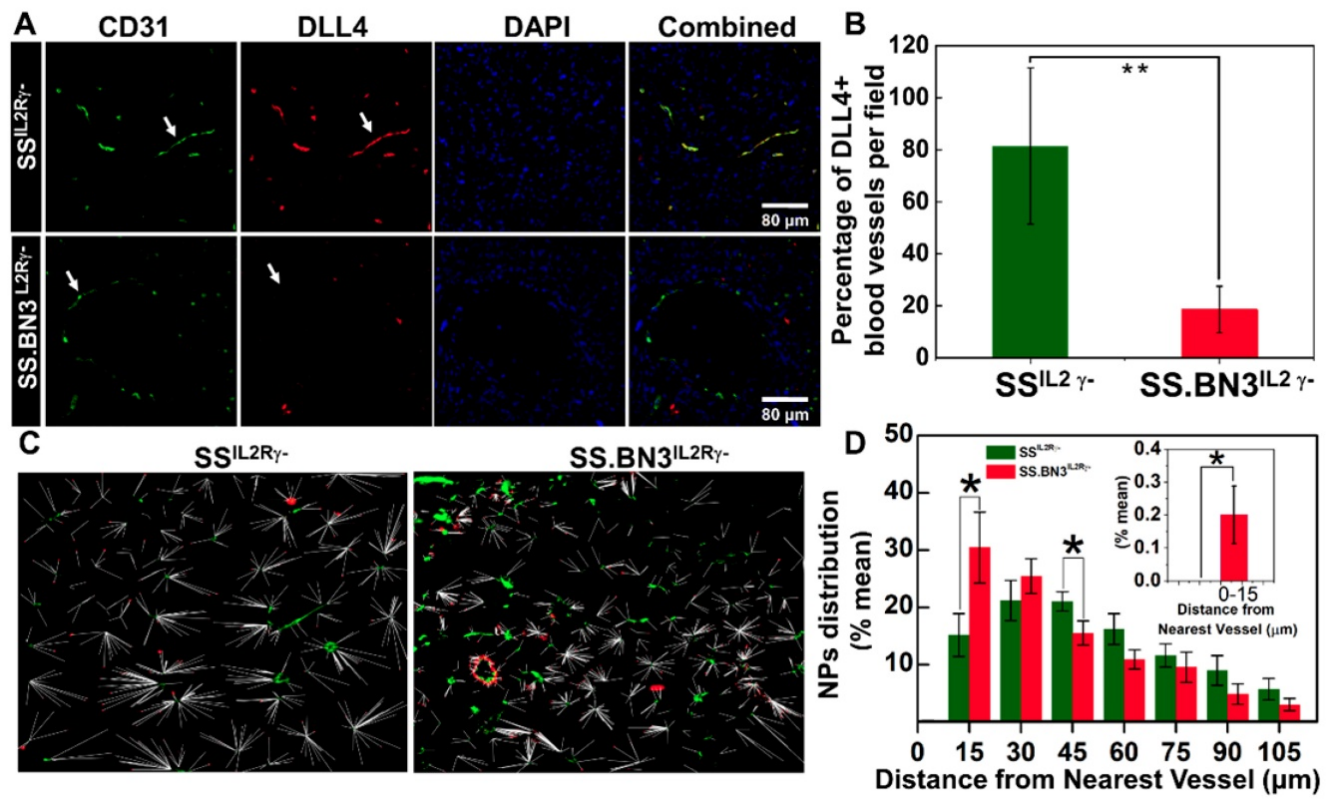
Distribution pattern and Quantitative analysis of TNPs. (A) Histology of tumor sections extracted from SS^IL2Rγ-^ and SS.BN3^IL2Rγ-^ rats systemically injected with TNPs. DAPI stains cell nucleus in blue; Alexa Fluor594 in combination with CD31 stains blood vessels in green; and FITC in combination with DLL4 staining in blood vessels in red. The three channels (DAPI, Alexa Fluor594 and FITC) are overlaid. Images were acquired at X40 magnification. Scale bar, 80 µm. (B) Quantification of the percentage of DLL4+ blood vessels in tumor sections extracted from SS^IL2Rγ-^ and SS.BN3^IL2Rγ-^ rats. DLL4 is co-localized with CD31+ tumor blood vessels and is downregulated in SS.BN3 tumors compared with SS^ IL2Rγ-^. (C) Distribution patterns of TNPs relative to vasculature. Merged dark field images of TNPs (red) and fluorescent images of blood vessels (green) from the same region of tumor of SS^IL2Rγ-^ (n = 5) and SS.BN3^IL2Rγ-^ (n = 5). Each white line represents the distance of each NP from the nearest blood vessel. (D) Quantitative evaluation of TNPs distance from the nearest blood vessels. This analysis confirmed that in SS.BN3^IL2Rγ-^ tumors ~31% TNPs adhere to tumor vessels or located near 30 µm distance as compared with 15% in SS^IL2Rγ-^ rats (*P* = 0.04). No TNPs are located near 15 µm distance in SS^IL2Rγ-^ tumors. The inset shows the quantitative distribution of TNPs located at 0-15 µm distance from the blood vessel. *Significant difference between distribution of TNPs in SS^IL2Rγ-^ and SS.BN3^IL2Rγ-^ determine by paired t-test.

**Vascular differences determine NPs distribution in tumor tissues.** Since the TNPs uptake was lower in SS.BN3^IL2Rγ-^ compared with SS^IL2Rγ-^ (**Figure 2A-B**), yet paradoxically TGI was significantly greater in SS.BN3^IL2Rγ-^ (**Figure 3A-F**) without an appreciable difference in ablative temperatures (**Figure 3G-H**), we hypothesized that the inherited TME vascular heterogeneity associated with DLL4 expression likely altered distribution of TNPs within the tumors. To test this hypothesis, 231^LUC+^ triple negative breast cancer tumors that were orthotopically implanted in SS.BN3^IL2Rγ-^ and SS^IL2Rγ-^ rats were collected at 10 days post-implantation and analyzed for DLL4-positive blood vessel density by immunofluorescent staining and for the distribution of TNPs by dark field imaging. Compared with SS^IL2Rγ-^ tumors, the density of DLL4-positive blood vessels decreased by 60% in triple negative breast cancer implanted in SS.BN3^IL2Rγ-^ rats (**Figure 5A-B**). These results confirmed our previous results obtained using IHC, DCE-MRI, micro-CT, and optical imaging indicating reduced DLL4 expression and concomitant non-functional angiogenesis in SS.BN3^IL2Rγ-^-hosted tumors 25, 26.

TNPs were visualized along with immunofluorescence imaging via dark-field microscopy in which TNPs in the tumor sections of 231^LUC+^ cells implanted in SS^IL2Rγ-^ and SS.BN3^IL2Rγ-^ appear as bright spots due to the enhanced light scattering 37. These dark field images were overlaid with immunofluorescence images of blood vessels and nuclear staining acquired from the same field. The distribution pattern of TNPs indicated that in SS.BN3^IL2Rγ-^ TNPs concentrated within or near the blood vessels, as compared with SS^IL2Rγ-^ tumor tissues (**Figure S5**). For further evaluation of the distribution patterns, the distance of each NP from the nearest vessel was determined from the merged images of NPs and fluorescence images of blood vessels. The representative merged images with the distance of each NP from the nearest blood vessel for SS^IL2Rγ-^ and SS.BN3^IL2Rγ-^ tumor tissues are shown in **Figure 5C**. For the analysis, the numbers of NPs within the vessels and within incremental 15 µm contours around the blood vessels were determined. These were converted to percentages by dividing the number of NPs in each region by the total number of NPs in each image within the region of 100 µm from the corresponding vessel segment. This analysis indicated that significantly more TNPs are located within the 15-30 µm of the blood vessels in tumor tissues from SS.BN3^IL2Rγ-^ hosts (*P* = 0.04; **Figure 5D**). Comparatively, in SS^IL2Rγ-^ tumor tissues significantly more TNPs are located in the region of 45-60 µm from the blood vessels (*P* = 0.05; **Figure 5D**). Moreover, no TNPs were detected from 0-15 µm from CD31- or DLL4-stained blood vessels in SS^IL2Rγ-^ tumor sections (**Figure 5D, inset**). These images indicate that aberrant and dysfunctional vasculature of tumors in SS.BN3^IL2Rγ-^ hosts are responsible for retention of approximately twice the number of NPs within 30 µm of blood vessels in SS.BN3^IL2Rγ-^ tumors relative to SS^IL2Rγ-^ tumors, and this causes significantly higher photothermal damage to 231^LUC+^ tumor vasculature in SS.BN3^IL2Rγ-^ strain during nanoparticle-mediated laser ablation.

**Figure 6:**
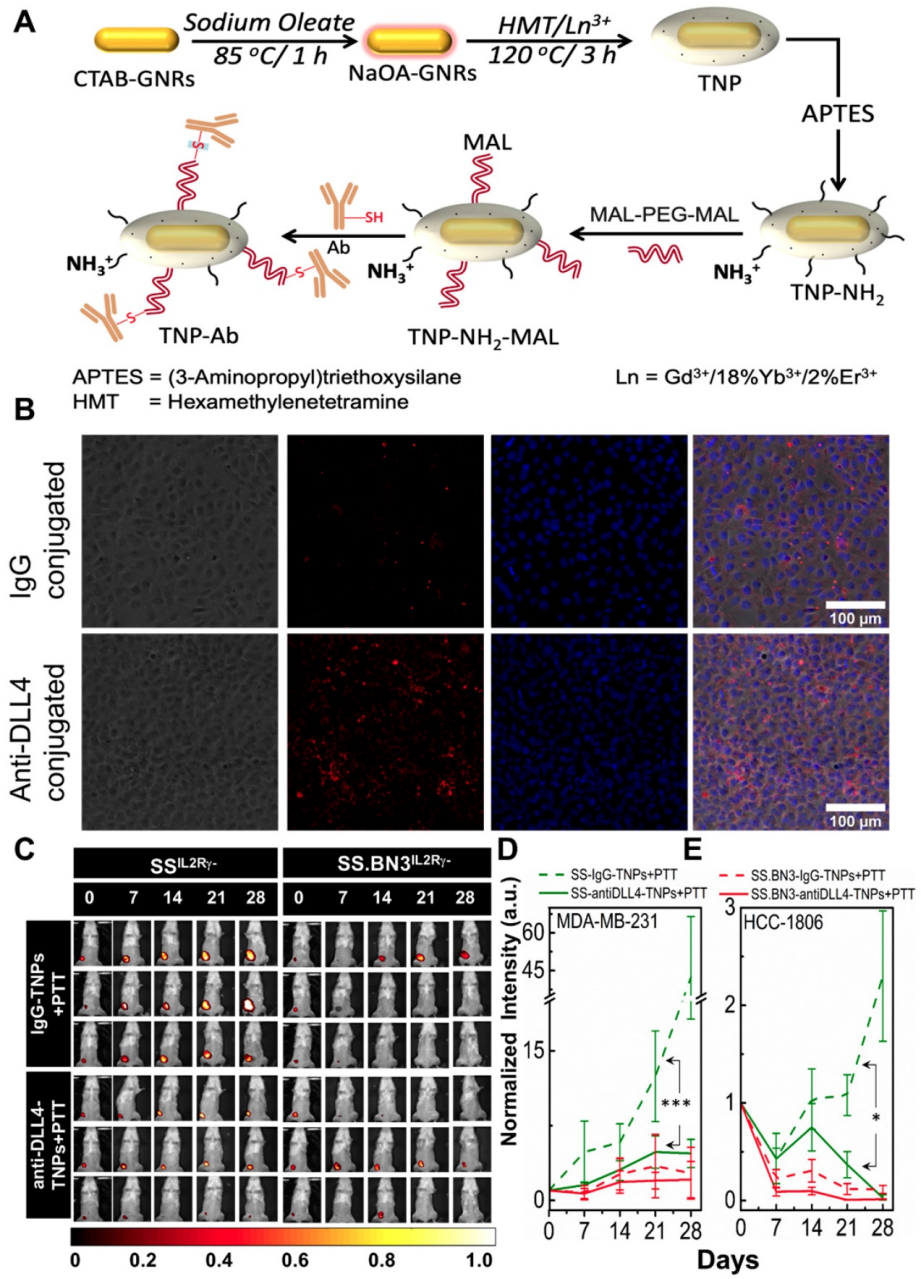
Conjugation of anti-DLL4 antibody to TNPs, evaluation of SS^IL2Rγ-^, and SS.BN3^IL2Rγ-^ strains response to photothermal therapy after the injection of DLL4 targeted TNPs. SS^IL2Rγ-^ and SS.BN3^IL2Rγ-^ rats were injected with IgG-conjugated and anti-DLL4 conjugated TNPs and followed by PTT. (A) Schematic for the synthesis of antibody (anti-DLL4/IgG) functionalized TNPs. (B) Distribution patterns of TNPs conjugated with IgG and anti-DLL4 in endothelial cells derived from heart of SS rats. Phase Contrast images of cells, Dark field images of TNPs, DAPI stains cell nucleus in blue. The three channels (DAPI, Phase Contrast and Dark Field) are overlaid. Images were acquired at X20 magnification. Scale bar, 100 µm. (C) Representative images of IgG-conjugated-TNPs and anti-DLL4-conjugated-TNPs treated SS^IL2Rγ-^ and SS.BN3^IL2Rγ-^ rats. SS^IL2Rγ-^ rats injected with IgG-TNPs (n=5) experienced a continuous increase of bioluminescence, while all SS.BN3^IL2Rγ-^ (n=5) and SS^IL2Rγ-^ (n=5) rats injected with anti-DLL4 conjugated TNPs rats experienced tumor inhibition after laser treatment. Rats were followed for 4 weeks after treatment. (D) The luciferase signal in all groups of SS^IL2Rγ-^ and SS.BN3^IL2Rγ-^rats was normalized to the signal before treatment. The normalized luciferase signal from SS^IL2Rγ-^and SS.BN3^IL2Rγ-^ rats implanted with HCC1806 tumors. SS^IL2Rγ-^ rats injected with IgG-TNPs (n=4) experienced a continuous increase of bioluminescence, while SS.BN3^IL2Rγ-^(n=4) injected with IgG-TNPs and both SS.BN3^IL2Rγ-^(n=5) and SS^IL2Rγ-^(n=5) rats injected with anti-DLL4 conjugated TNPs rats experienced tumor inhibition after laser treatment. Rats were followed for 4 weeks after treatment. *Significant difference in bioluminescence of SS^ IL2Rγ-^ treated with anti-DLL4 conjugated TNPs (*P* < 0.002) with respect to SS^IL2Rγ-^ treated with IgG-conjugated TNPs was determined by fit linear regression model. All the data are shown as the mean±s.e.

**Theranostic anti-DLL4 conjugated Nanoparticles.** Antibody functionalized TNPs were synthesized as depicted in **Figure 6A**. The average size of the nanoparticles was sub-100 nm measured using Transmission electron microscopy (**Figure S1A**). Maleimide functionalized nanoparticles had the charge of ~ 7.6 mV with a hydrodynamic diameter of ~235 nm. On functionalization with anti-DLL4 and IgG (control) on the surface of the nanoparticles, charge slightly decreased to 3.56 mV and 4.16 mV, respectively. Change in both hydrodynamic diameter and zeta potential of the nanoparticles during the conjugation process are depicted in **Figure S1B**. An ELISA method was used to evaluate antibody (anti-DLL4) conjugation on the surface of the TNPs (**Figure S1C**). To further validate the successful conjugation procedure of anti-DLL4-conjugated TNPs, we used SS^IL2Rγ-^ heart derived endothelial cells and determined distribution of these nanoparticles in contrast with IgG-conjugated-TNPs by dark field microscopy as depicted in **Figure 6B**. Significant enhancement in dark field intensity in anti-DLL4 coated group compared with IgG group confirmed the higher specificity of anti-DLL4 TNP to the rat endothelial cells compared with control IgG antibody.

**PTT with anti-DLL4-conjugated TNPs inhibits tumor growth in SS^IL2Rγ-^ (DLL4-high) as SS.BN3^IL2Rγ-^ (DLL4-low).** To assess the impact of anti-DLL4-conjugated TNPs on SS^IL2Rγ-^ and SS.BN3^IL2Rγ-^ rats were implanted with MDA-MB-231(231^LUC+^) triple negative breast cancer cells in the inguinal mammary fat pad. Rats were randomized into two groups per strain: group 1 (anti-DLL4-conjugated TNPs +laser; n = 6) and group 2 (IgG-conjugated TNPs +laser; n = 5). Analysis of tumor growth inhibition (TGI) by bioluminescent imaging revealed strong TGI in SS^IL2Rγ-^ rats treated by anti-DLL4-conjugated TNPs followed by PTT (**Figure 6C-D**). Tumor regression was similar in SS.BN3^IL2Rγ-^ rats whether treated by anti-DLL4-conjugated TNPs or IgG-conjugated TNPs followed by PTT (**Figure 6D**), however, strong differences in TGI were observed in SS^IL2Rγ-^ (DLL4-high strain) rats when treated by DLL4-conjugated TNPs vs control IgG-TNPs. These differences were statistically significant (*P* = 0.002) after adjusting for other covariates, via multilinear regression.

To further verify, the impact of anti-DLL4-conjugated TNPs on SS^IL2Rγ-^ and SS.BN3^IL2Rγ-^ rats were implanted with another triple negative breast cancer cells HCC-1806 (1806^RLUC+^) in the inguinal mammary fat pad. Rats were randomized into two groups per strain: group 1 (anti-DLL4-conjugated TNPs +laser; n = 5) and group 2 (IgG-conjugated TNPs +laser; n = 4). Analysis of tumor growth inhibition (TGI) by bioluminescent imaging verified similar TGI was observed in SS^IL2Rγ-^ rats bearing 1806^rluc+^ tumors and treated by anti-DLL4-conjugated TNPs as compared to SS^IL2Rγ-^ rats treated with control IgG-TNPs (**Figure 6E** and **Figure S6**). This data again demonstrates that DLL4 expression plays a role in PTT therapy resistance and targeting DLL4 strongly modifies the therapeutic efficacy of PTT with TNPs.

## Discussion

Nanomedicine literature supports a direct dynamic relationship between the tumor vasculature dysfunction and NP uptake and retention in solid tumors 18, 38, 39. However, tumor vasculature dysfunction or the EPR effects is not constant and varies with tumor type, stage, and a number of underlying factors such as the tumor stroma, lymphatics and inflammatory cytokines 19, 40, 41. It is important to understand the effect of these vascular variability factors on NP uptake and therapy response, especially in emerging personalized medicine paradigms, both for selecting patient specific nanotherapies, as well as to modulate nanotherapy regimens with complimentary vascular therapies such as DLL4 blockade 42, 43 in patients with therapy resistance. Despite the underlying importance of vascular variability, the inherited factors which determine the heterogeneity of EPR, and thus predict the success or failure of tumor-targeted nanomedicine, have not been studied extensively. One of the reasons for this inadequate knowledge is the limited availability of relevant animal models of cancer. To address these knowledge gaps, Song *et al.*, evaluated the therapeutic efficacy of NPs in models with variable TME and tumor types by using genomically-validated and engineered mouse models of basal-like (C3(1)/SV40 T/t-antigen) and claudin-low [T11/TP53/(T11)] mammary tumors 44.

Although, this study provided evidence that broader TME and/or tumor characteristics affect NP delivery and therapeutic efficacy, the specific effect of EPR differences in the host stroma could not be evaluated in these genetic engineered mouse models. Here, we used germline genetic variants in the host TME which differed in both tumor vascular density and function, while maintaining identical tumor cell implants. The consomic and congenic models reported in this manuscript are unique and to date, no such preclinical model, which perfectly reiterates differences in germline-driven vascular microenvironment in otherwise identical tumors, has been reported to study the uptake and response of nanomedicine.

Recent studies have emphasized the role of imaging in identifying patients likely to favorably respond to nanomedicine. Clinical dynamic MRI imaging of breast cancer patients has indicated a strong correlation of EPR dependent contrast wash-in and wash-out profiles with eventual cancer progression and therapy response 45. Barbier and Jean-Luc Coll *et al.* reported dynamic contrast-enhanced MRI (DCE-MRI) and steady-state vessel size index MRI to quantitatively determine vascular parameters of the tumor and the TME and identified vascular permeability and tumor blood volume fraction as predictive markers of “effective EPR” 46-49. We reported previously that SS.BN3^IL2Rγ-^-hosted tumors with low-DLL4 expression in the tumor-associated vasculature has tumor permeability and contrast retention behavior similar to the therapy responsive disease observed in clinic 25. The results in this study demonstrate that these DLL4-driven differences in tumor vascular function directly impact nanoparticle distribution and response to photothermal therapy, thus DCE-MRI or other vascular function imaging alone or in combination with genomic profiling can be developed to identify tumors with favorable prognosis to nanoparticle therapies 25, 26.

The MR visible TNPs reported here allow direct monitoring of NP deposition in tumors to allow the determination of optimal therapy time-points. Our MRI findings agreed with the quantitative ICP-MS results to verify tumor uptake performed after systemic delivery of both AuNRs and TNPs (**Figure 2**). Our previous and present results suggested that morphological and functional differences in tumor vasculature in SS^IL2Rγ-^ and SS.BN3^IL2Rγ-^ consomics 26. The vasculature of SS.BN3 have higher permeability but lower perfusion but high DLL4 expressing SS^IL2Rγ-^ hosted tumors retain higher overall levels of NPs at 4 h time point due to a better perfusion effect dominated at the initial time-point. Although this delivery advantage was not significant enough to impact the temperature rise in tumors, and counterintuitively the SS^IL2Rγ-^ hosted tumors had a much worse therapy response compared with lower DLL4 expressing and dysfunctional angiogenesis-bearing tumors in SS.BN3^IL2Rγ-^ hosts. Approximately 60-80% of SS.BN3^IL2Rγ-^ animals that were treated with AuNRs or TNPs and laser had complete responses, whereas none of SS^ IL2Rγ-^ strains showed strong growth inhibition (**Figure 3**). Surprisingly, this observation could not be attributed to differences in post-PTT ablative temperatures within tumors implanted in either strain, nor could it be linked to differences in total NP accumulation between the tumors. Rather, we found that the differences in therapeutic response between the SS^IL2Rγ-^ and SS.BN3^IL2Rγ-^ host strains were due to differing intratumoral distribution of NPs, which were likely attributed to the inherited differences in vascular patterning between the two strains (**Figure 1 and Figure S2**). The effect of DLL4 levels in the tumor microenvironment on uptake and therapy response was independent of the NP configuration in terms of shape and sizes for the two variants tested in this manuscript: (1) PEGylated Au nanorods with dimensions 10 by 40 nm, hydrodynamic radius: 80 nm, charge -9 mV, and (2) PEGylated TNPs with Gd2O3 coating on Au nanorods resulting in oblong spheroid shape of size 75 nm, hydrodynamic radius 235 nm, +7.6 mV. We note that both rod and spheroid shapes of differing nanoparticle dimensions, similar tumor uptake and therapy response differences were observed in DLL4-high and low tumors as illustrated in **Figure 3A** (TNPs) and **Figure S2** (Au-NRs). Thus, we expect that for nanoparticles sizes of 50 - 100 nm, the role of DLL4 microenvironment on the uptake and therapy response is independent of size and these findings should hold for other plasmonic nanostructures in these size regimes 50-52.

As mentioned above, we and others have demonstrated that DLL4 is a key regulator of vascular patterning and angiogenesis 28-35, which can lead to inherited differences in vascular patterning. Here, we demonstrated using *ex vivo* 3D microCT images that vascular structure, density, and thickness of vasculature differ in the SS.BN3^IL2Rγ-^ host (low-DLL4) compared with the SS^IL2Rγ-^ host (high-DLL4), which was further supported by immunofluorescence imaging of tumor sections (**Figure 1 and Figure S2**). The increased branching and tortuosity of tumor blood vessels in the SS.BN3^IL2Rγ-^ hosted (low-DLL4) tumors fits with previously reported data demonstrating that pharmacological inhibition of DLL4 attenuates tumor growth and progression by eliciting nonproductive angiogenesis (i.e., a higher density of poorly functioning vasculature) 31-34. Despite the well-known role of DLL4 in vascular patterning, the impact of inherited differences in vascular patterning on PTT with NPs was not previously explored. By overlaying dark field imaging of NPs with immunofluorescent imaging of tumor blood vessels, we found marked differences in intratumoral distribution of NPs in tumors growing in the SS.BN3^IL2Rγ-^ host (low-DLL4) compared with the SS^IL2Rγ-^ host (high-DLL4) (**Figure 5**). SS^IL2Rγ-^ hosted tumors with higher reported perfusion 26 had consistently higher uptake of NPs at the therapy time-points of 4-24 h, yet the nanoparticles were on the average distributed away from the vasculature compared to SS.BN3^IL2Rγ-^ hosts. We postulate that the greater concentration of NPs near the blood vessels in the SS.BN3^IL2Rγ-^ host tumors provides a distinct advantage during PTT by obliterating tumor blood vessels, whereas this effect is dampened in SS^IL2Rγ-^ tumors with more diffuse distribution of NPs. Since tumor blood vessels are critical for delivering oxygen and nutrients to support tumor cell viability, we conclude that host TME modifiers of vascular patterning, such as DLL4, can dramatically impact the intratumoral distribution of NPs and, in turn, the responsiveness of tumors to PTT with NPs. Optical Imaging techniques have demonstrated that microvascular remodeling and hemodynamic changes occur during tumor growth and inflammation 53, 54. These studies clearly demonstrate that tumor growth requires a dense vascular system to supply nutrients and oxygen to tissues, and without this vascular support tumor growth falters and a complete vascular shutdown can eradicate tumors, as seen in therapy response in SS.BN3^IL2Rγ-^ rats. This complete eradication of tumor can be in response to photothermal therapy or photodynamic effect. The interesting observation regarding limited penetration of nanoparticles in SS.BN3^IL2Rγ-^ (within the distance of 15-30 µm) will require further in depth study. On the basis of evidence in literature about the likely reasons for limited penetration in DLL4-low tumors. We believe that this difference could be due to absence of α-smooth muscle actin (SMA) in SS.BN3^IL2Rγ-^ vasculature. DLL-4-Notch signaling controls cell fate in endothelial cells and also plays a regulatory role in pericyte formation 55. In Ewing sarcoma mouse models, it has been demonstrated that inhibition of DLL4 expression results in reduced numbers of bone marrow derived pericytes/ vascular smooth muscle cells (vSMCs) and less functional vessels than tumors of control-treated mice 55. In bladder cancer, it has been reported that compared to approximately 60% of DLL4-negative vessels, 98% of DLL4-positive tumor vessels are surrounded by pericytes and vSMCs cells 34. Pericytes and vSMCs, collectively referred to as mural cells, lend support and contractility to blood vessels 56. We hypothesize that pericytes and vSMCs are absent or less in SS.BN3^IL2Rγ-^ blood vessels which results in localization of nanoparticles only at a distance of 15-30 µm of the blood vessels.

Further, we verified our hypothesis and injected anti-DLL4 conjugated TNPs (0.75-1 mg/Kg of DLL4 antibody) in SS^IL2Rγ-^ strain with MDA MB 231 and HCC 1806 tumors. PTT with anti-DLL4 conjugated TNPs demonstrated positive therapeutic outcome and tumor growth inhibition similar to SS.BN3^IL2Rγ-^ (DLL4-low) strain **(Figure 6).** These results confirmed previous preclinical conclusions that vascular targeted NPs increase the efficacy of PTT 57, radiotherapy 58, 59 and delivery of chemotherapeutics to tumor tissues by exerting cytotoxic effects on both endothelial and tumor cells. Targets on the tumor endothelium/blood vessels such as peptides, proteins, antibodies, genes, siRNAs and miRNAs that suppress different aspects of endothelial cell behaviors or simultaneously eradicate tumor cells have been conjugated to NPs and assessed for vascular targeted therapies. Among these, αβ integrins 60, 61, VEGR-VEGFR-2 62, endoglin 63, and nucleolin 64 have been successfully studied to target NPs to tumor associated endothelium. Recently, several groups have independently identified DLL4, a member of the Notch/Delta family located to the tumor endothelium, as a potential target for vascular targeted therapy of tumors 31, 35, 65. We previously reported that in SS.BN3^IL2Rγ-^ hosts, increased vascular branching and density coincided with decreased expression of DLL4 25. We confirm in this work that either low expression of DLL4 or DLL4-targeted nanoparticles can result in improved therapeutic responses. Several anti-DLL4 monoclonal antibodies have successfully demonstrated broad preclinical antitumor activity, with multiple anti-DLL4 molecules currently investigated as potential cancer therapeutics 33, 42, 66-68. Despite successful results, clinical trials with DLL4-targeted antibodies and various preclinical studies with rats reported that intravenous injections of anti-DLL4 at 10 mg/kg every 3 days for a total of five doses results in pathological changes of liver and vascular neoplasms of skin, heart, and lungs 69. We demonstrated that DLL4-targeted nanoparticles with a small fraction of anti-DLL4 dose (0.75-1 mg/Kg of DLL4 antibody) compared with anti-DLL4 monotherapy provides an effective therapeutic option for patients whose genetic variants promote upregulated DLL4 expression in the tumor vasculature and nanoparticle mediated blockade of DLL4 may not cause dose-related liver and vascular toxicities. In summary, we demonstrate that DLL4 is an important component of the Notch signaling pathway and mediates tumor growth through self-renewal of tumor initiating cells and vascular development. The high expression of DLL4 in endothelial cells of ovarian 70, breast 71, nasopharyngeal 12 and renal cancer 43 has been reported. We have assessed inherited DLL4 expression in endothelial cells modulates EPR effects in tumors, and molecular targeting of endothelial DLL4 via nanoparticles is an effective nanomedicine strategy. This strategy can be an effective treatment in most of the tumor models that overexpress endothelial DLL4. Since, tumor angiogenesis is governed by cross-talk between VEGF and Delta-Notch pathway, in some cases, effective tumor regression can be obtained by nanoparticles bounded with dual-specific antibodies targeting both DLL4 and VEGF as reported in glioblastoma 72.

In conclusion, our study highlights that cancer is a highly heterogeneous disease and success of nanomedicine depends critically on inherited tumor vascular microenvironment genes, independent of tumor type. These host genes such as DLL4 can determine individual differences in uptake, distribution of nanoparticles and response of nanoparticle mediated therapies. Here, we showed that such differences can be identified and modeled in animal systems, and therapy resistant hosts can be specifically targeted for increased nanomedicine efficacy. Personalized nanomedicine can be developed by mapping genetic differences such as DLL4 which are involved in vessel branching and maturation and play an important role in nanomedicine therapy response. Thus patients with high endothelial DLL4 expression can be selected for treatment with anti-Dll4 targeted nanoparticles, vs patients with low-DLL4 expression, where PEGylated nanoparticles will provide sufficient therapy response.

## Methods

**Synthesis and Characterization of NPs.** TNPs were synthesized by the method as previously published 36. TNPs composed of NIR plasmon-resonant core (GNRs) and a Gd (III) inorganic layer as the shell. Au core was first synthesized using a seed-mediated growth process 73 followed by sodium oleate coating at 80^°^C for 1 h. Uniform Gd(III) shell was achieved in the presence of hexamethylenetetramine at 120 ^°^C for 3 h. In typical synthesis process, Au seed particles were prepared by adding 0.5 mL of a 5 mM HAuCl_4_ solution (HAuCl_4_·3H_2_O, >99.9%, Fluka) to 5 mL of distilled water and 5 mL of 0.2 M CTAB (Hexadecyltrimethylammonium bromide, >98%, Sigma-Aldrich) solution. The resulting solution was stirred, and ∼0.6 mL of ice-cold 0.1 M NaBH_4_ (Sodium borohydride, >98%, Sigma-Aldrich) was added. The seed particles were kept at room temperature. For the growth of GNRs, 1.14 mL of AgNO_3_ (ACS reagent, >99%, Sigma- Aldrich) solution (0.1 M) and 1.125 mL of 1.2 M HCl (VWR Analytical) were added to 450 mL of CTAB (0.2 M), and the resulting solution was vortexed. Then, 90 mL of 0.005 M HAuCl_4_ was added and mixed with 55.5 mL of 0.01 M ascorbic acid (AA, 99%, Sigma). The color of the resulting solution became dark yellow first and immediately turned colorless. Then, 750 μL of Au seed solution was added and mixed for 20 s, and GNRs were grown for 12 h. Then, CTAB-stabilized GNRs were centrifuged at 12,000 rpm for 15 min and redispersed in distilled water. Centrifugation was performed twice, and the GNRs were dispersed in 0.02 M NaOA and heated at 80 °C for 1 h. The final concentration of NaOA-GNR was adjusted to 5 × 10^11^ NP/mL.

TNPs were prepared by growing a Gd(III) shell on NIR-resonant GNR-NaOA. For a typical synthesis of TNPs, 150 mL of GNR-NaOA (10^11^ NP/mL) was added to 450 mL of distilled water and vortexed. Then, 1.5 mL of 0.1 M Hexamethylenetetramine (HMT, >99%, Sigma-Aldrich) and 4.5 mL of Gd(III)-nitrate precursor having Yb/Er = 18:2% (0.01 M, 99.9%, Aldrich) were added, vortexed, and sonicated for 30 min using a sonication probe. The resulting solution was heated at 120 °C for 3 h and then cooled to room temperature. The TNPs were left undisturbed overnight, and the transparent supernatant was carefully removed. The resulting solution was centrifuged at 3000 rpm for 5 min and redispersed in distilled water. Centrifugation was repeated two times, and the solution was redispersed in 10 mL of dimethylformamide (DMF) solvent. Then, 100 μL of 10% APTES was added, and the mixture was vortexed for 3 min, followed by heating the solvent at 80 °C for 12 h. Excess APTES was removed by centrifugation. In this manner, amine-functionalized TNPs (TNP-NH_2_) were prepared. To obtain neutral TNP-mPEG, ∼10^13^ NPs/mL was dispersed in 20 mL of DMF solvent with 0.1 mM of mPEG5k-COOH (Nanocs, New York, NY), 0.12 mM of benzotriazol-1-yl-oxy-tris(dimethylamino)phosphonium hexafluorophosphate (BOP reagent, 97%, Aldrich) reagent, and 15 μL of triethylamine (99%, Sigma-Aldrich). The resulting mixture was stirred at room temperature for 12 h. The obtained PEGylated NPs was dispersed in PBS and concentrated to the desired concentration and stored in a refrigerator for further use. The surface-modified TNPs were studied by dispersing NPs in 10 mM NaCl solution, and the ζ- potential was measured. For antibody conjugation, TNP-NH_2_ nanoparticles were reacted with ~100-folds excess of MAL-PEG5k-MAL (Nanocs, New York, NY), and the reaction was continued for 4 h according to the schematic depicted in **Figure 6A**. During this process, some -NH_2_ groups were replaced with maleimide groups. Antibodies (Ab = anti-DLL4/IgG, HMD4-1, Invitrogen, Carlsbad, CA, USA) initially reacts with TCEP (tris (2-carboxyethyl) phosphine for 5 min and then PBS added to slowdown the disulphide reduction reaction. These Ab's reacts with maleimide coated TNPs overnight at 4 ^°^C and redispersed in ultrapure water after three times washing, particles concentrated to ~10^13^ nanoparticles/mL and stored at 4 ^°^C. The absorption spectra of TNPs were measured using an Infinite 200 PRO (Tecan, Mannedorf, Switzerland) spectrophotometer. The hydrodynamic size via dynamic light scattering and ζ potential of TNPs were measured using a Malvern Zetasizer Nano ZS (Malvern Instruments, United Kingdom) operated at 25°C. For confirmatory experiments with non-theranostic PEG-coated AuNRs reported in supplementary data, dense hydrophilic PEGylated AuNRs with 40 nm × 10 nm dimensions and longitudinal plasmon resonances at 810 nm (D12M-808-Bulk) were obtained from Nanopartz, a division of Concurrent Analytical, Inc.

**Cell Culture and Triple Negative Breast Cancer Xenografts.** Firefly- luciferase expressing MDA-MB-231^LUC+^ and mCherry-renilla luciferase expressing HCC1806 cells were maintained in DMEM media (Sigma), supplemented with 10% FBS (Gibco) and 1% penicillin and streptomycin (Lonza) and incubated in 5% CO_2_ at 37°C as described in the literature. All *in vivo* studies were conducted in accordance with institutional guidelines and under approved IACUC protocols at the Medical College of Wisconsin. These Luciferase expressing cells (6 x 10^6^) in 50% Matrigel were orthotopically implanted into the mammary fat pads (MFP) of 4- to 6-week-old female SS^IL2Rγ-^, SS.BN3^IL2Rγ-^ and the 3 SS.BN3^IL2Rγ-^ congenic strains JQ^IL2Rγ-^, ND^IL2Rγ-^ and MX^IL2Rγ-^25. Tumors were treated after 10 days of implantation at an approximate size of 600 mm^3^, which was consistent across all rat strains.

**Photothermal Therapy.** To study the effect of photothermal therapy of TNPs on breast cancer, 32 (16 SS^IL2Rγ-^ and 16 SS.BN3^IL2Rγ-^) rats were randomized into 3 study groups for each strain. Group-1 (n = 4; saline+laser) was injected with saline and laser treated, the second Group-2 (n = 4; only TNPs, no laser treatment) and Group-3 (n = 8; TNPs+laser) was tail vein injected with 1 μL/g of 10^13^ TNPs/mL and 4 h later treated with laser. For laser treatment, a class-IV diode laser (DioMed, D 15 *Plus*) was employed and a uniformly expanded beam covering tumor circumference was generated by coupling the fiber optic (1" diameter, lens at 20 cm far from animal) carrying the laser light from the diode with a plano-convex lens. Average power at skin surface was kept constant at 1.65 W/cm^2^ for all laser-treated groups. For the AuNRs-PTT experiment, 16 (8 of SS^IL2Rγ-^ and 8 of SS.BN3^IL2Rγ-^) were randomized into 2 groups for each strain. Group 1 (n = 3; saline+laser) was injected with saline, and Group 2 (n = 5; AuNRs+Laser) was injected with AuNRs via tail vein followed by laser treatment at 24 h, which was determined to be the optimal time point for AuNRs accumulation in tumors via ICP-MS studies. The injected dose was 1 μL/g (weight of the animal) of 10^13^ AuNRs/mL stock concentration. The laser ablation procedure was identical to the TNPs-groups. Post-therapy all rats were followed *via* bioluminescence imaging up to 4 weeks or death (euthanized), whichever was earlier. Animals were euthanized due to tumor burden according to IACUC guidelines. There was no obvious toxic effect of TNPs on the animals as acute toxicity and clearance of systemically delivered TNPs in healthy male and female rats has been rigorously verified in our previous publication 36.

**AuNRs and TNPs Bio distribution studies.** After 10 days of tumor implantation, 18 (9 SS^IL2Rγ-^ and 9 SS.BN3^IL2Rγ-^) rats were randomized into 3 groups with 3 rats per group. The injected dose was 1 μL/g (weight of the animal) of 10^13^ AuNRs/mL. NPs were injected via tail vein. Four, 24 and 72 h after injection, animals were sacrificed and organs including brain, heart, lung, liver, spleen, gut, kidney, blood and tumor were collected. The organs were washed in PBS and stored at -80°C until further investigation. For ICP-MS sample preparation, all tissues were dissolved in nitric acid (HNO_3_; 90%) and hydrogen peroxide (H_2_O_2_; 10%) and heated at 80°C until completely dissolved. 3% (by volume) of the dissolved tissue was diluted with distilled water and used to determine the metal content per gram of tissue by inductively coupled plasma mass spectrometry (ICP-MS, PerkinElmer) analysis. For TNPs, MRI reported biodistribution was verified quantitatively via identical ICP-MS studies with 3 rats each of SS^ IL2Rγ-^ and SS.BN3^ IL2Rγ-^ strains bearing xenografted tumors, and sacrificed post 24 h time point MRI imaging. The injected dose was 1 μL/g (weight of animal) of 10^13^ TNPs/mL, were injected via tail vein.

**Micro-CT.** Tumor bearing rats were perfused with saline containing 5 U/mL of heparin, followed by systemic injection of the Microfil casting agent (Flow Tech, Inc., Carver, MA). The tumor was excised and further processed, as described previously 25. The 3D CT data sets were acquired over 230-ms integration time with 2048 proj/180, 28.9-mm-diameter field of view at nominal resolution of 27 µm using a Triumph SPECT/CT scanner. An X-ray source of voltage 65 kVp and a beam current 170 μA were used. The spatial graph view and vessel fraction of tumor analysis was performed using Amira 6.2.0 (Amira 6.2; TGS, Berlin, Germany).

**Histopathology.** Tumors were washed in PBS, frozen sectioned and immunostained with antibodies against blood vessel marker, CD31 (BD Biosciences, San Jose, CA, USA), DLL4 (R&D Systems, Minneapolis, MN, USA), and the nucleus was stained with DAPI (Vector Lab, Inc., Burlingame, CA) as described previously 25. The details of the antibodies are provided in **Table S1**. Dark field microscopy and fluorescence microscopy of tumor sections were conducted with Nikon Eclipse E600 fluorescent microscope with a 20x and 40 x objectives. The NPs are visualized due to enhanced light scattering properties of Au. The illuminated signals from each dark field image were merged with a fluorescence image of blood vessels from the same field of view to determine NPs distribution in both consomics. The distance of each NP from the nearest blood vessel was quantitatively analyzed in the MATLAB environment.

**Image Processing and Statistical Analysis.** Image processing, data analysis and PCA were performed in MATLAB (Matlab 2016b, Mathworks, Nattick MA, USA) software with Image Processing Toolbox and custom scripts. Survival Analysis was performed with Prism software (Prism 7, GraphPad Software, La Jolla California USA).

## Supplementary Material

Supplementary figures and table.

## References

[1] Barenholz Y (2012). Doxil(R)-the first FDA-approved nano-drug: lessons learned. J Control Release.

[2] Gradishar WJ, Tjulandin S, Davidson N, Shaw H, Desai N, Bhar P (2005). Phase III trial of nanoparticle albumin-bound paclitaxel compared with polyethylated castor oil-based paclitaxel in women with breast cancer. J Clin Oncol.

[3] Prabhakar U, Maeda H, Jain RK, Sevick-Muraca EM, Zamboni W, Farokhzad OC (2013). Challenges and key considerations of the enhanced permeability and retention effect for nanomedicine drug delivery in oncology. Cancer Res.

[4] Gradishar WJ, Krasnojon D, Cheporov S, Makhson AN, Manikhas GM, Clawson A (2009). Significantly longer progression-free survival with nab-paclitaxel compared with docetaxel as first-line therapy for metastatic breast cancer. J Clin Oncol.

[5] Petersen GH, Alzghari SK, Chee W, Sankari SS, La-Beck NM (2016). Meta-analysis of clinical and preclinical studies comparing the anticancer efficacy of liposomal versus conventional non-liposomal doxorubicin. J Control Release.

[6] Von Hoff DD, Mita MM, Ramanathan RK, Weiss GJ, Mita AC, LoRusso PM (2016). Phase I Study of PSMA-Targeted Docetaxel-Containing Nanoparticle BIND-014 in Patients with Advanced Solid Tumors. Clin Cancer Res.

[7] Munster P, Krop IE, LoRusso P, Ma C, Siegel BA, Shields AF (2018). Safety and pharmacokinetics of MM-302, a HER2-targeted antibody-liposomal doxorubicin conjugate, in patients with advanced HER2-positive breast cancer: a phase 1 dose-escalation study. Br J Cancer.

[8] Jones SW, Roberts RA, Robbins GR, Perry JL, Kai MP, Chen K (2013). Nanoparticle clearance is governed by Th1/Th2 immunity and strain background. J Clin Invest.

[9] Riley RS, Day ES (2017). Gold nanoparticle-mediated photothermal therapy: applications and opportunities for multimodal cancer treatment. Wiley Interdiscip Rev Nanomed Nanobiotechnol.

[10] Efficacy Study of AuroLase Therapy in Subjects With Primary and/or Metastatic Lung Tumors. https://ClinicalTrials.gov/show/NCT01679470

[11] Xu Q, Wan J, Bie N, Song X, Yang X, Yong T (2018). A Biomimetic Gold Nanocages-Based Nanoplatform for Efficient Tumor Ablation and Reduced Inflammation. Theranostics.

[12] Zhang JX, Cai MB, Wang XP, Duan LP, Shao Q, Tong ZT (2013). Elevated DLL4 expression is correlated with VEGF and predicts poor prognosis of nasopharyngeal carcinoma. Med Oncol.

[13] Zhang L, Yang XQ, Wei JS, Li X, Wang H, Zhao YD (2019). Intelligent gold nanostars for in vivo CT imaging and catalase-enhanced synergistic photodynamic & photothermal tumor therapy. Theranostics.

[14] Zhang W, Ding X, Cheng H, Yin C, Yan J, Mou Z (2019). Dual-Targeted Gold Nanoprism for Recognition of Early Apoptosis, Dual-Model Imaging and Precise Cancer Photothermal Therapy. Theranostics.

[15] Fedele C, Tothill RW, McArthur GA (2014). Navigating the challenge of tumor heterogeneity in cancer therapy. Cancer Discov.

[16] Zhao B, Hemann MT, Lauffenburger DA (2014). Intratumor heterogeneity alters most effective drugs in designed combinations. Proc Natl Acad Sci U S A.

[17] Collins FS, Varmus H (2015). A new initiative on precision medicine. N Engl J Med.

[18] Matsumura Y, Maeda H (1986). A new concept for macromolecular therapeutics in cancer chemotherapy: mechanism of tumoritropic accumulation of proteins and the antitumor agent smancs. Cancer Res.

[19] Nel A, Ruoslahti E, Meng H (2017). New Insights into "Permeability" as in the Enhanced Permeability and Retention Effect of Cancer Nanotherapeutics. ACS Nano.

[20] Bort G, Lux F, Dufort S, Cremillieux Y, Verry C, Tillement O (2020). EPR-mediated tumor targeting using ultrasmall-hybrid nanoparticles: From animal to human with theranostic AGuIX nanoparticles. Theranostics.

[21] Dhaliwal A, Zheng G (2019). Improving accessibility of EPR-insensitive tumor phenotypes using EPR-adaptive strategies: Designing a new perspective in nanomedicine delivery. Theranostics.

[22] Goos J, Cho A, Carter LM, Dilling TR, Davydova M, Mandleywala K (2020). Delivery of polymeric nanostars for molecular imaging and endoradiotherapy through the enhanced permeability and retention (EPR) effect. Theranostics.

[23] Flister MJ, Bergom C (2018). Genetic Modifiers of the Breast Tumor Microenvironment. Trends Cancer.

[24] Flister MJ, Endres BT, Rudemiller N, Sarkis AB, Santarriaga S, Roy I (2014). CXM: a new tool for mapping breast cancer risk in the tumor microenvironment. Cancer Res.

[25] Flister MJ, Tsaih SW, Stoddard A, Plasterer C, Jagtap J, Parchur AK (2017). Host genetic modifiers of nonproductive angiogenesis inhibit breast cancer. Breast Cancer Res Treat.

[26] Jagtap J, Sharma G, Parchur AK, Gogineni V, Bergom C, White S (2018). Methods for detecting host genetic modifiers of tumor vascular function using dynamic near-infrared fluorescence imaging. Biomed Opt Express.

[27] Suchting S, Freitas C, le Noble F, Benedito R, Breant C, Duarte A (2007). The Notch ligand Delta-like 4 negatively regulates endothelial tip cell formation and vessel branching. Proceedings of the National Academy of Sciences of the United States of America.

[28] Hellstrom M, Phng LK, Hofmann JJ, Wallgard E, Coultas L, Lindblom P (2007). Dll4 signalling through Notch1 regulates formation of tip cells during angiogenesis. Nature.

[29] Siekmann AF, Lawson ND (2007). Notch signalling limits angiogenic cell behaviour in developing zebrafish arteries. Nature.

[30] Lobov IB, Renard RA, Papadopoulos N, Gale NW, Thurston G, Yancopoulos GD (2007). Delta-like ligand 4 (Dll4) is induced by VEGF as a negative regulator of angiogenic sprouting. Proc Natl Acad Sci U S A.

[31] Noguera-Troise I, Daly C, Papadopoulos NJ, Coetzee S, Boland P, Gale NW (2006). Blockade of Dll4 inhibits tumour growth by promoting non-productive angiogenesis. Nature.

[32] Scehnet JS, Jiang W, Kumar SR, Krasnoperov V, Trindade A, Benedito R (2007). Inhibition of Dll4-mediated signaling induces proliferation of immature vessels and results in poor tissue perfusion. Blood.

[33] Xu Z, Wang Z, Jia X, Wang L, Chen Z, Wang S (2016). MMGZ01, an anti-DLL4 monoclonal antibody, promotes nonfunctional vessels and inhibits breast tumor growth. Cancer Lett.

[34] Ridgway J, Zhang G, Wu Y, Stawicki S, Liang WC, Chanthery Y (2006). Inhibition of Dll4 signalling inhibits tumour growth by deregulating angiogenesis. Nature.

[35] Hoey T, Yen WC, Axelrod F, Basi J, Donigian L, Dylla S (2009). DLL4 blockade inhibits tumor growth and reduces tumor-initiating cell frequency. Cell Stem Cell.

[36] Parchur AK, Sharma G, Jagtap JM, Gogineni VR, LaViolette PS, Flister MJ (2018). Vascular Interventional Radiology-Guided Photothermal Therapy of Colorectal Cancer Liver Metastasis with Theranostic Gold Nanorods. ACS Nano.

[37] Ayala-Orozco C, Urban C, Knight MW, Urban AS, Neumann O, Bishnoi SW (2014). Au nanomatryoshkas as efficient near-infrared photothermal transducers for cancer treatment: benchmarking against nanoshells. ACS Nano.

[38] Maeda H (2015). Toward a full understanding of the EPR effect in primary and metastatic tumors as well as issues related to its heterogeneity. Adv Drug Deliv Rev.

[39] Maeda H, Wu J, Sawa T, Matsumura Y, Hori K (2000). Tumor vascular permeability and the EPR effect in macromolecular therapeutics: a review. J Control Release.

[40] Bae YH, Park K (2011). Targeted drug delivery to tumors: myths, reality and possibility. J Control Release.

[41] Fang J, Nakamura H, Maeda H (2011). The EPR effect: Unique features of tumor blood vessels for drug delivery, factors involved, and limitations and augmentation of the effect. Adv Drug Deliv Rev.

[42] Jia X, Wang W, Xu Z, Wang S, Wang T, Wang M (2016). A humanized anti-DLL4 antibody promotes dysfunctional angiogenesis and inhibits breast tumor growth. Sci Rep.

[43] Miles KM, Seshadri M, Ciamporcero E, Adelaiye R, Gillard B, Sotomayor P (2014). Dll4 blockade potentiates the anti-tumor effects of VEGF inhibition in renal cell carcinoma patient-derived xenografts. PLoS One.

[44] Song G, Darr DB, Santos CM, Ross M, Valdivia A, Jordan JL (2014). Effects of tumor microenvironment heterogeneity on nanoparticle disposition and efficacy in breast cancer tumor models. Clin Cancer Res.

[45] Daniel BL, Yen YF, Glover GH, Ikeda DM, Birdwell RL, Sawyer-Glover AM (1998). Breast disease: dynamic spiral MR imaging. Radiology.

[46] Beaumont M, Lemasson B, Farion R, Segebarth C, Remy C, Barbier EL (2009). Characterization of tumor angiogenesis in rat brain using iron-based vessel size index MRI in combination with gadolinium-based dynamic contrast-enhanced MRI. J Cereb Blood Flow Metab.

[47] Karageorgis A, Dufort S, Sancey L, Henry M, Hirsjarvi S, Passirani C (2016). An MRI-based classification scheme to predict passive access of 5 to 50-nm large nanoparticles to tumors. Sci Rep.

[48] Pannetier NA, Debacker CS, Mauconduit F, Christen T, Barbier EL (2013). A simulation tool for dynamic contrast enhanced MRI. PLoS One.

[49] Ng TSC, Garlin MA, Weissleder R, Miller MA (2020). Improving nanotherapy delivery and action through image-guided systems pharmacology. Theranostics.

[50] Khoury CG, Vo-Dinh T (2008). Gold Nanostars For Surface-Enhanced Raman Scattering: Synthesis, Characterization and Optimization. J Phys Chem C Nanomater Interfaces.

[51] Stoerzinger KA, Hasan W, Lin JY, Robles A, Odom TW (2010). Gold Nanopyramids Assembled into High-Order Stacks Exhibit Increased SERS Response. J Phys Chem Lett.

[52] Tangeysh B, Moore Tibbetts K, Odhner JH, Wayland BB, Levis RJ (2015). Triangular gold nanoplate growth by oriented attachment of Au seeds generated by strong field laser reduction. Nano Lett.

[53] Stamatelos SK, Kim E, Pathak AP, Popel AS (2014). A bioimage informatics based reconstruction of breast tumor microvasculature with computational blood flow predictions. Microvasc Res.

[54] Rege A, Thakor NV, Rhie K, Pathak AP (2012). In vivo laser speckle imaging reveals microvascular remodeling and hemodynamic changes during wound healing angiogenesis. Angiogenesis.

[55] Stewart KS, Zhou Z, Zweidler-McKay P, Kleinerman ES (2011). Delta-like ligand 4-Notch signaling regulates bone marrow-derived pericyte/vascular smooth muscle cell formation. Blood.

[56] Hellstrom M, Gerhardt H, Kalen M, Li X, Eriksson U, Wolburg H (2001). Lack of pericytes leads to endothelial hyperplasia and abnormal vascular morphogenesis. J Cell Biol.

[57] Day ES, Zhang L, Thompson PA, Zawaski JA, Kaffes CC, Gaber MW (2012). Vascular-targeted photothermal therapy of an orthotopic murine glioma model. Nanomedicine (Lond).

[58] Berbeco RI, Ngwa W, Makrigiorgos GM (2011). Localized dose enhancement to tumor blood vessel endothelial cells via megavoltage X-rays and targeted gold nanoparticles: new potential for external beam radiotherapy. Int J Radiat Oncol Biol Phys.

[59] Koonce NA, Levy J, Hardee ME, Jamshidi-Parsian A, Vang KB, Sharma S (2015). Targeting Artificial Tumor Stromal Targets for Molecular Imaging of Tumor Vascular Hypoxia. PLoS One.

[60] Bogorad RL, Yin H, Zeigerer A, Nonaka H, Ruda VM, Zerial M (2014). Nanoparticle-formulated siRNA targeting integrins inhibits hepatocellular carcinoma progression in mice. Nat Commun.

[61] Hamilton AM, Aidoudi-Ahmed S, Sharma S, Kotamraju VR, Foster PJ, Sugahara KN (2015). Nanoparticles coated with the tumor-penetrating peptide iRGD reduce experimental breast cancer metastasis in the brain. J Mol Med (Berl).

[62] Wicki A, Rochlitz C, Orleth A, Ritschard R, Albrecht I, Herrmann R (2012). Targeting tumor-associated endothelial cells: anti-VEGFR2 immunoliposomes mediate tumor vessel disruption and inhibit tumor growth. Clin Cancer Res.

[63] Chen F, Hong H, Zhang Y, Valdovinos HF, Shi S, Kwon GS (2013). In vivo tumor targeting and image-guided drug delivery with antibody-conjugated, radiolabeled mesoporous silica nanoparticles. ACS Nano.

[64] Wu J, Song C, Jiang C, Shen X, Qiao Q, Hu Y (2013). Nucleolin targeting AS1411 modified protein nanoparticle for antitumor drugs delivery. Mol Pharm.

[65] Fischer M, Yen WC, Kapoun AM, Wang M, O'Young G, Lewicki J (2011). Anti-DLL4 inhibits growth and reduces tumor-initiating cell frequency in colorectal tumors with oncogenic KRAS mutations. Cancer Res.

[66] Smith DC, Eisenberg PD, Manikhas G, Chugh R, Gubens MA, Stagg RJ (2014). A phase I dose escalation and expansion study of the anticancer stem cell agent demcizumab (anti-DLL4) in patients with previously treated solid tumors. Clin Cancer Res.

[67] Chiorean EG, LoRusso P, Strother RM, Diamond JR, Younger A, Messersmith WA (2015). A Phase I First-in-Human Study of Enoticumab (REGN421), a Fully Human Delta-like Ligand 4 (Dll4) Monoclonal Antibody in Patients with Advanced Solid Tumors. Clin Cancer Res.

[68] Huang J, Hu W, Hu L, Previs RA, Dalton HJ, Yang XY (2016). Dll4 Inhibition plus Aflibercept Markedly Reduces Ovarian Tumor Growth. Mol Cancer Ther.

[69] Yan M, Callahan CA, Beyer JC, Allamneni KP, Zhang G, Ridgway JB (2010). Chronic DLL4 blockade induces vascular neoplasms. Nature.

[70] Hu W, Lu C, Dong HH, Huang J, Shen DY, Stone RL (2011). Biological roles of the Delta family Notch ligand Dll4 in tumor and endothelial cells in ovarian cancer. Cancer Res.

[71] Jubb AM, Soilleux EJ, Turley H, Steers G, Parker A, Low I (2010). Expression of vascular notch ligand delta-like 4 and inflammatory markers in breast cancer. Am J Pathol.

[72] Li Y, Hickson JA, Ambrosi DJ, Haasch DL, Foster-Duke KD, Eaton LJ (2018). ABT-165, a Dual Variable Domain Immunoglobulin (DVD-Ig) Targeting DLL4 and VEGF, Demonstrates Superior Efficacy and Favorable Safety Profiles in Preclinical Models. Mol Cancer Ther.

[73] Nikoobakht B, El-Sayed MA (2003). Preparation and growth mechanism of gold nanorods (NRs) using seed-mediated growth method. Chemistry of Materials.

