# Supplementary material for: Heritable modifiers of the tumor microenvironment influence nanoparticle uptake, distribution and response to photothermal therapy
Source: Theranostics. 2020 Apr 6;10(12):5368–83. doi: 10.7150/thno.41171 (PMC7196309; doi:10.7150/thno.41171)
Supplement: Supplementary file 1 — Supplementary figures and table. [file thnov10p5368s1.pdf]

SUPPORTING INFORMATION

Heritable Modifiers of the Tumor Microenvironment Influence Nanoparticle Uptake, Distribution and Response to Photothermal Therapy

Gayatri Sharma,[1] Jaidip M. Jagtap,[1] Abdul K. Parchur,[1*] Venkateswara R. Gogineni,[2] Sophia Ran,[3,4] Carmen Bergom,[5] Sarah B. White,[2] Michael J. Flister,[6,7] and Amit Joshi[1, 2,✉]*

[1]Department of Biomedical Engineering, Medical College of Wisconsin, Milwaukee, WI, USA, [2]Department of Radiology, Medical College of Wisconsin, Milwaukee, WI, USA, [3]Simmons Cancer Institute, Southern Illinois University School of Medicine, Springfield, IL, USA, [4]Department of Medical Microbiology, Immunology, and Cell Biology, Southern Illinois University School of Medicine, Springfield, IL, USA. [5]Department of Radiation Oncology, Medical College of Wisconsin, Milwaukee, WI, USA, [6]Department of Physiology Medical College of Wisconsin, Milwaukee, WI, USA, [7]Genomic Sciences and Precision Medicine Center, Medical College of Wisconsin, Milwaukee, WI 53226, USA

[*]Both authors have contributed equally

✉ Corresponding Author: Amit Joshi, PhD
Associate Professor of Biomedical Engineering and Radiology
Medical College of Wisconsin
Translational & Biomedical Research Center | 2nd Floor, Rm C2930
8701 Watertown Plank Road | Milwaukee, WI 53226-3548
Phone: (414) 955-7588 | Fax: (414) 805-8282 | Cell: (281) 224-9584
Email: ajoshi@mcw.edu

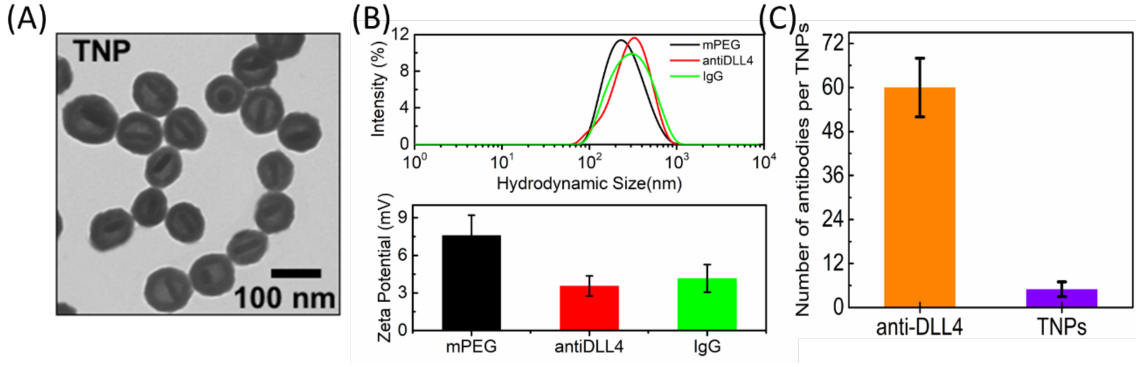

Figure S1. (A) TEM image, (B) Hydrodynamic size, and zeta potential of TNPs (C) Quantification of the number of antibodies on anti-DLL4 conjugated TNPs and control (only-TNPs). Values depicted from control-TNPs is a negligible signal in comparison to anti-DLL4 conjugated TNPs.

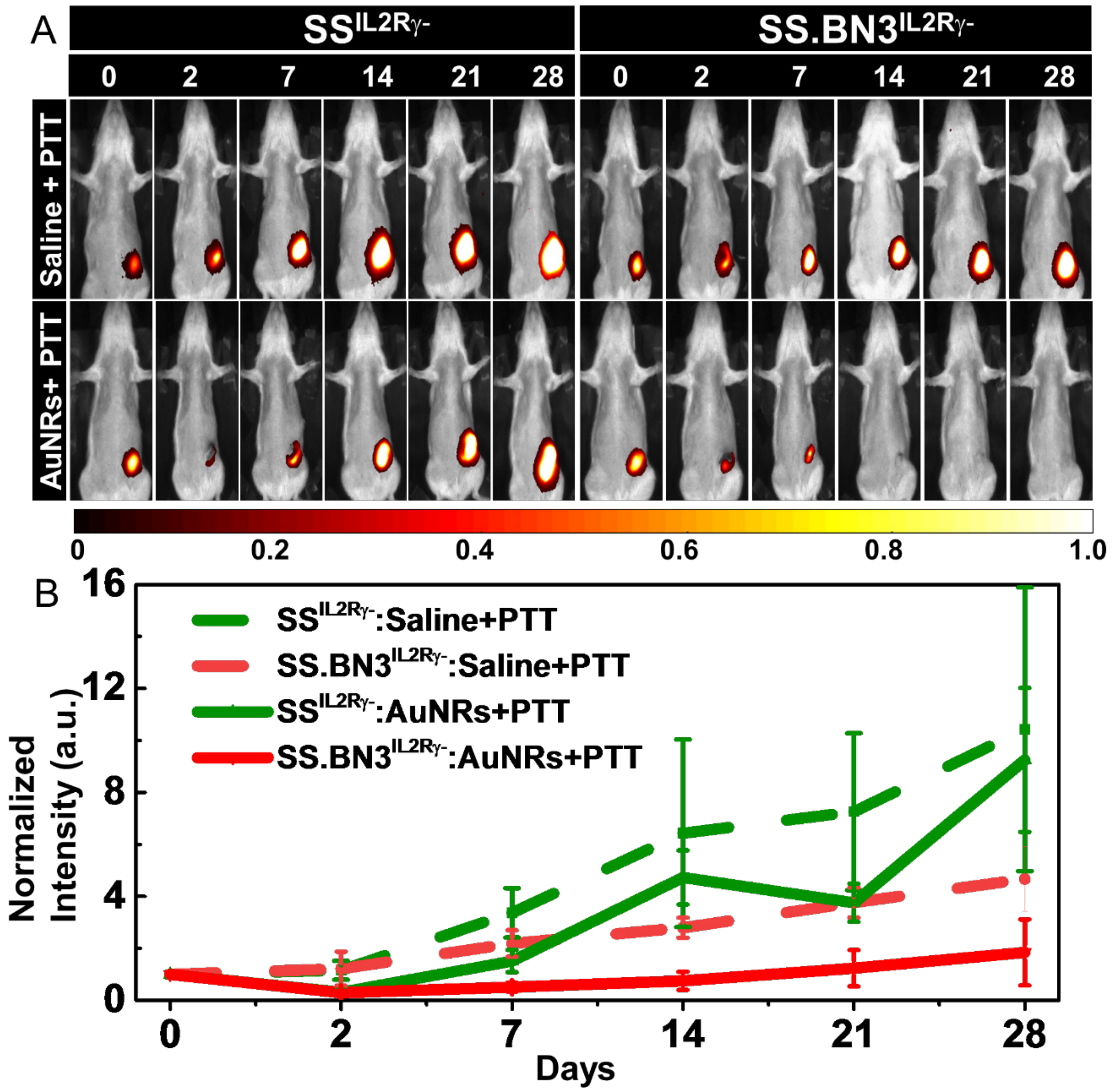

Figure S2. (A) Representative images of saline and AuNRs treated SS $^{IL2R\gamma-}$ (n=3 and n=5) and SS.BN3$^{IL2R\gamma-}$ (n=3 and n=5) rats. AuNRs injected SS $^{IL2R\gamma-}$ and SS.BN3 $^{IL2R\gamma-}$ rats when treated with laser at first experienced loss of bioluminescence but later after a week SS$^{IL2R\gamma-}$ rats experienced increase in bioluminescence while SS.BN3$^{IL2R\gamma-}$ rats experienced complete loss of tumor. Saline injected SS $^{IL2R\gamma-}$ and SS.BN3$^{IL2R\gamma-}$ rats when treated with laser experienced a continuous increase of bioluminescence. Rats were followed for 4 weeks after treatment (B) Mean bioluminescence in the tumor with standard error. The bioluminescence in all groups was normalized to the signal before treatment.

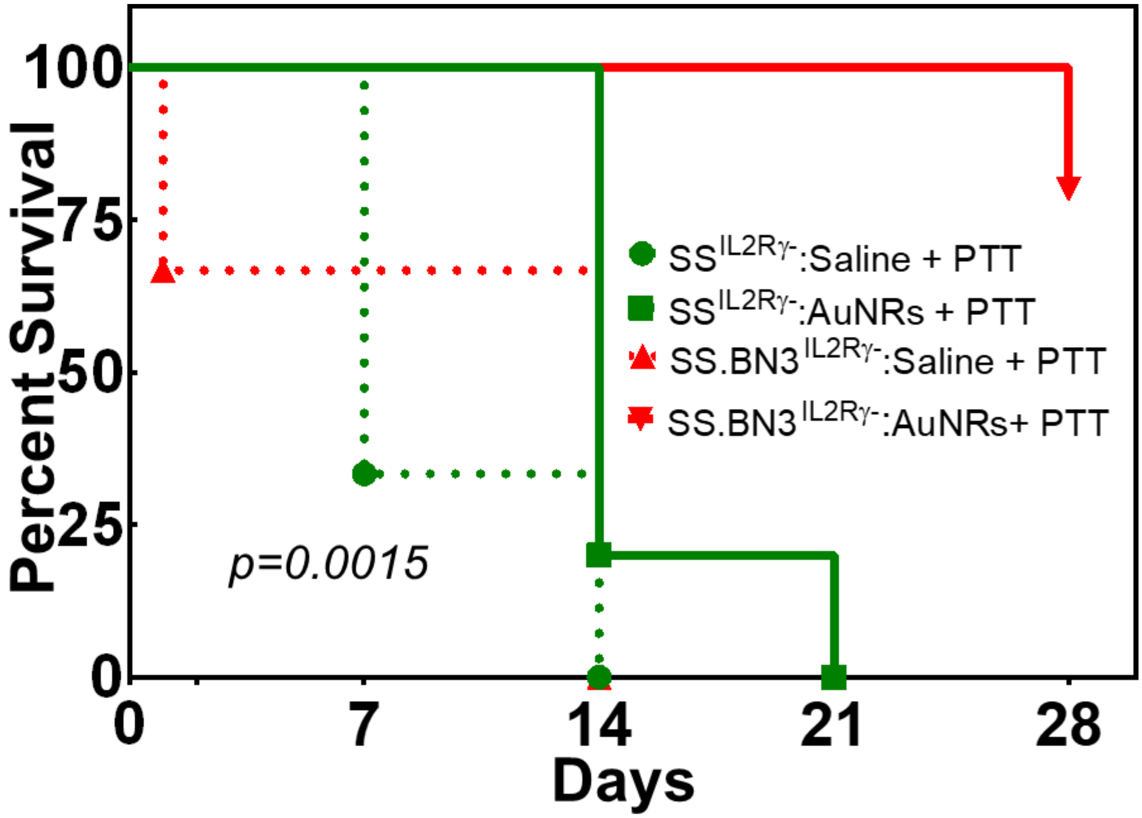

Figure S3. Survival curves of tumor-bearing SS.BN3$^{IL2R\gamma-}$ treated with saline and AuNRs followed by 808-nm NIR laser irradiation for 5 min with 1.65 W/cm^2 laser power covering 2 cm^2 areas. Rats treated with AuNRs and laser responded better, and trend difference was statistically significant (*P*=0.015).

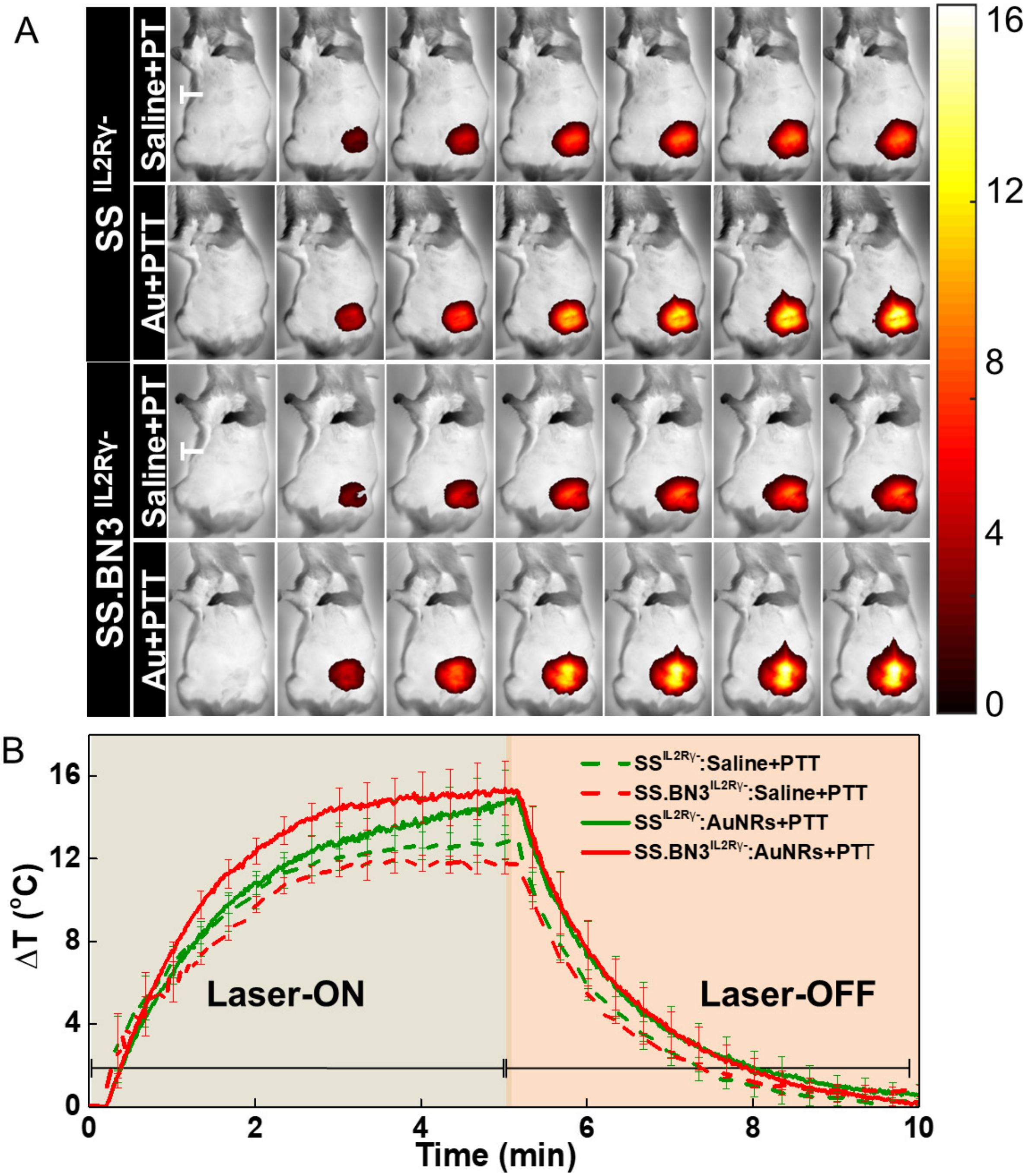

Figure S4. (A) *In vivo* Photothermal Therapy of 231^{LUC+} implanted $SS^{IL2R\gamma-}$ and $SS.BN3^{IL2R\gamma-}$ rats after AuNRs and saline are injected. FLIR thermal images of 231^{LUC+} implanted $SS^{IL2R\gamma-}$ and $SS.BN3^{IL2R\gamma-}$ rats acquired after 4 h of saline and AuNRs systemic injection, irradiated by an 808-nm NIR laser for 5 min with 1.65 W/cm^2 laser power covering 2 cm^2 areas. (B) Temperature kinetics of $SS^{IL2R\gamma-}$ and $SS.BN3^{IL2R\gamma-}$ rats during 5 min irradiation by an 808-nm NIR laser with 1.65 W/cm^2 laser power covering 2 cm^2 area, followed by cooling for 5 min. Temperature change (ΔT) is calculated by subtracting the surface temperature at the starting time point (37°C).

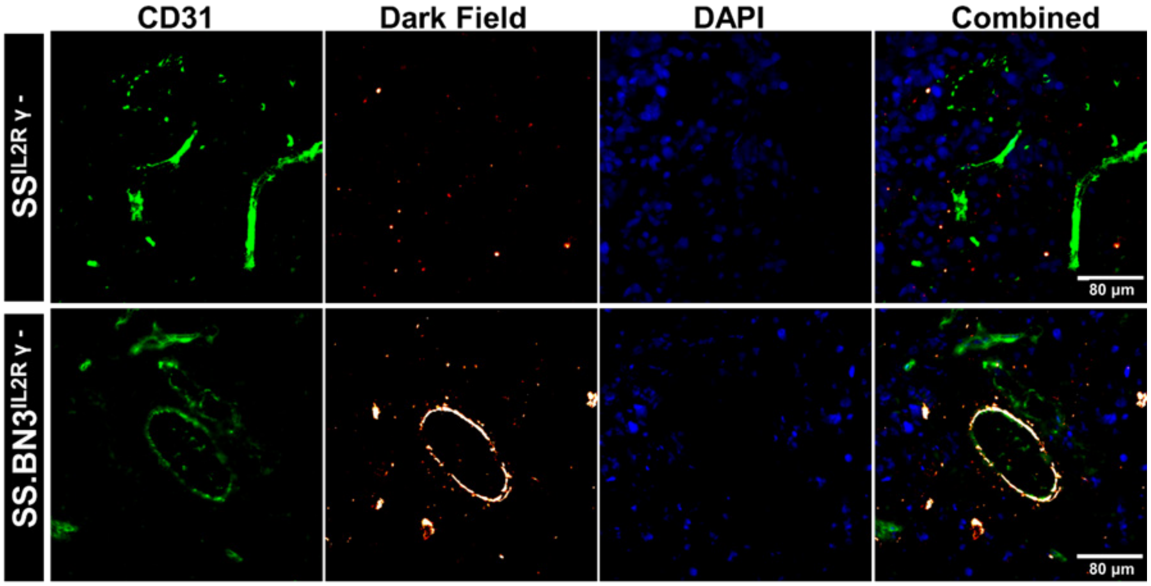

Figure S5. Vasculature differences and distribution patterns of TNPs relative to vasculature. Fluorescent images of blood vessels and merged dark field images of TNPs from the same region of tumor of $SS^{IL2R\gamma-}$ (n=5) and $SS.BN3^{IL2R\gamma-}$ (n=5). DAPI stains cell nucleus in blue; Alexa Fluor594 in combination with CD31 stains blood vessels in green. The three channels (DAPI, Alexa Fluor594 and Dark Field) are overlaid. Images were acquired at X40 magnification. Scale bar, 80 um.

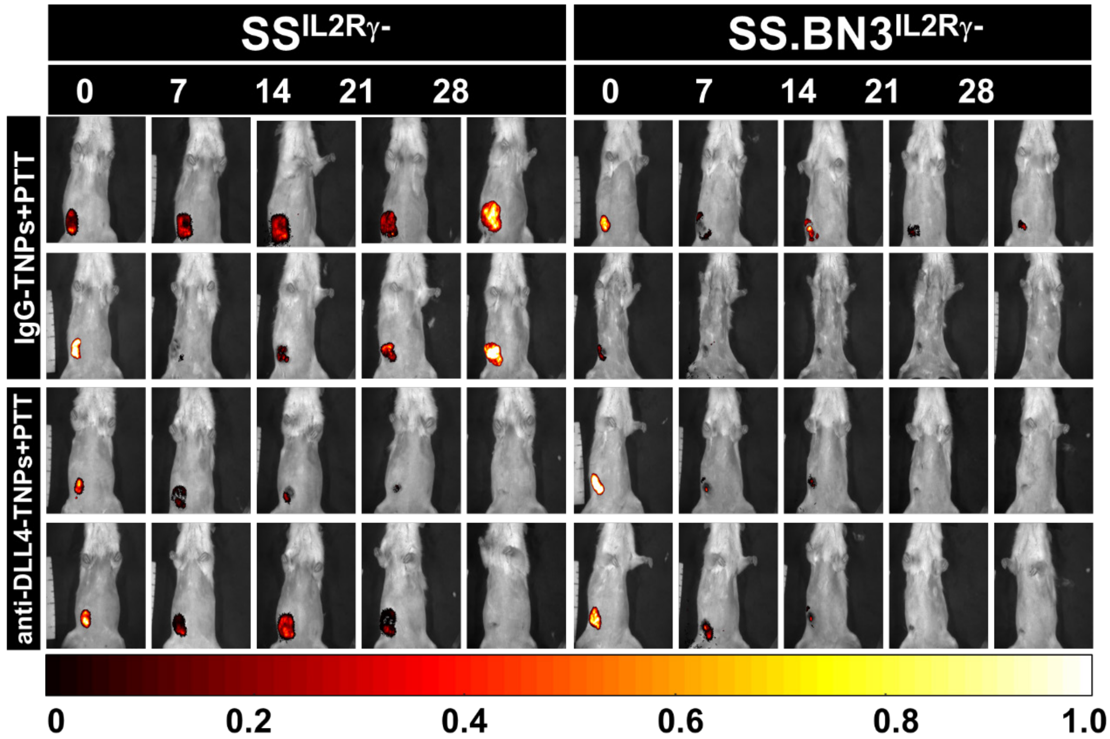

Figure S6. Representative images of IgG-conjugated-TNPs and anti-DLL4-conjugated-TNPs treated SS$^{IL2R\gamma-}$ and SS.BN3$^{IL2R\gamma-}$ rats with 1806^{RLUC+} breast cancer tumors. SS$^{IL2R\gamma-}$ rats injected with IgG-TNPs (n=4) experienced a continuous increase of bioluminescence, while all SS.BN3$^{IL2R\gamma-}$ (n=5) and SS$^{IL2R\gamma-}$ (n=5) rats injected with anti-DLL4 conjugated TNPs rats experienced tumor inhibition after laser treatment. Rats were followed for 4 weeks after treatment.

Table S1: Details of the antibodies

S No.	Primary	Clone/Catalog	Concentration
1	CD31	TLD-3A12	1:50
2	DLL4 (for Immunofluorescence)	AF1389	1:100
3	DLL4 (Functional grade for targeted therapy)	HMD4-1	
